# Exploration of Genomic Features and Volatile Flavor Production of Yeasts Isolated from Commercial Korean Rice Wine Makgeolli

**DOI:** 10.4014/jmb.2605.05009

**Published:** 2026-08-03

**Authors:** Su Jin Yoo, Jongbeom Kim, Yeon Ju Park, Haeyoung Park, Hye Yun Moon, Sang Wan Han, Seon Woo Song, Seong-Il Eyun, Che Ok Jeon, Hyun Ah Kang

**Affiliations:** Department of Life Science, Chung-Ang University, Seoul 06974, Republic of Korea

**Keywords:** *Saccharomyces cerevisiae*, Fermented rice wine, Whole-genome sequencing, Volatile flavors, 4-Vinylguaiacol

## Abstract

In this study, we identified and characterized yeast strains isolated from commercially available Korean rice wine, Kooksoondang Yetnal (KY), Haechang (HC), Yangchon Ureongii (YU), and Cheongju Sinseonju (CS) makgeolli products. ITS sequence analysis identified all the yeast isolates as *Saccharomyces cerevisiae*. Most makgeolli isolates exhibited better growth at 15°C than the reference strain *S. cerevisiae* S288C. Ploidy and whole-genome sequencing analyses of six representative strains revealed that all strains possessed diploid genomes. Phylogenetic analysis based on genome sequences indicated that all makgeolli strains clustered with the Asian-origin sake/ragi lineage. HC-CAU28, KY-CAU1, KY-CAU18, and YU-CAU13 were closely related to the Japanese sake strain K7, whereas CS-CAU12 and CS-CAU26 shared a common ancestor with the Chinese industrial bioethanol strain YJSH1 and Indonesian ragi strain Y9. Compared to S288C, the makgeolli isolates displayed similar volatile flavor profiles but produced higher levels of several flavor compounds, including acetoin, diacetyl, and benzaldehyde, consistent with higher expression of the corresponding biosynthetic genes. Notably, HC-CAU28, KY-CAU1, KY-CAU18, and YU-CAU13 did not produce a clove-like flavor 4-vinylguaiacol (4-VG), reflecting the presence of a nonsense mutation in their *FDC1* genes encoding ferulic acid decarboxylase involved in conversion of ferulic acid to 4-VG. The 4-VG production activity of the *fdc1* mutant strains was recovered by introducing *FDC1* overexpression vectors except YU-CAU13. These findings on flavor production capacity of commercial makgeolli yeast strains, based on genomic information and validated with genetic manipulation, would provide a foundation for developing yeast starters tailored for rice wine with optimized sensory and functional properties.

## Introduction

‘Makgeolli’, a Korean traditional alcoholic beverage (also known as ‘*takju*’ in Korea), is a type of rice wine typically prepared using steamed rice and nuruk, a natural fermentation starter [1]. The quality of such beverages in terms of alcohol (ethanol), flavor (organic acids and amino acids), and aroma (ester compounds and higher alcohols) is influenced mainly by the action of various bacteria, yeast, and filamentous fungi through the saccharification and fermentation process [2]. Several yeast species, including the conventional yeast *Saccharomyces cerevisiae* and various non-conventional yeasts found in natural nuruk, contribute to the production of various flavor compounds [3, 4]. However, as fermentation progresses, the heterogeneity of the yeast species in nuruk dramatically diminishes, and *S. cerevisiae* becomes the dominant microorganism [5]. This is due to the ability of *S. cerevisiae* to rapidly produce ethanol and tolerate high concentrations of alcohol in the fermentation process [6]. The microbial succession observed in nuruk exemplifies the broader challenges of spontaneous fermentation, which relies entirely on autochthonous microorganisms. During spontaneous fermentation, different species and strains of fungi, yeasts, and bacteria are promoted on natural starters at different intensities. Depletion of growth factors and unpredictable enzymatic effects can occur, making the quality control of wine products fermented by autochthonous yeasts quite difficult. Thus, induced fermentation using commercially produced active dried yeast as starters is predominant in the wine industry, despite the resulting narrow aromatic profiles and uniformity of wine products [7].

Several *S. cerevisiae* strains have been exploited for specific fermentation and industrial processes. Comparing genomes of *S. cerevisiae* strains from different origins, including natural and human-related isolates, helps to clarify the natural history of yeast populations. Furthermore, this identifies genomic elements governing the metabolic activities and physiological characteristics essential for biotechnological applications [8-11]. Studies analyzing human-related *S. cerevisiae* subpopulations have reported various independent and lineage-specific domestication events. One well-documented domesticated trait is the selection of beer yeasts for the production of 4-vinylguaiacol (4-VG), a phenolic compound with a smoke-like flavor that contributes markedly to the characteristic off-flavor. Thus, many industrial yeasts used for beer brewing have acquired loss-of-function mutations in the *PAD1* and/or *FDC1* genes involved in 4-VG production, resulting in a loss of the ability to produce 4-VG. Conversely, it is a selected trait for wheat and Belgian beer, and greatly contributes to their characteristic clove-like flavor [12, 13].

To obtain insights into the origin and flavor-producing capacity of key yeast strains used in industrial rice wine brewing, we investigated yeast strains isolated from various commercial makgeolli products in Korea, including Kooksoondang Yetnal (KY), Haechang (HC), Yangchon Ureongii (YU), and Cheongju Sinseonju (CS). We characterized the ploidy, genome structure, growth patterns, volatile flavor profiles, and 4-VG production of these strains, as these factors play crucial roles in shaping the alcohol content, taste, and aroma of makgeolli during saccharification and fermentation. Comparative genomic analysis provided valuable insights into the evolutionary origin of the makgeolli yeast isolates. Furthermore, the flavor-formation activities of the makgeolli yeast strains were validated using genomic data, transcript-level analysis, and genetic manipulation. This information will contribute to the development of yeast starters with enhanced flavor and functionality for traditional fermented alcoholic beverages.

## Materials and Methods

### Strains, Plasmids, and Culture Conditions

The yeast strains used in this study are listed in Table 1. The plasmids and primers used for recombinant yeast strains and quantitative reverse transcription PCR (qRT-PCR) are listed in Supplementary Table 1. All yeast strains were typically cultivated in a Yeast Extract-Peptone-Dextrose (YPD) medium (1% yeast extract, 2% Bacto peptone, 2% glucose) at 28°C. For isolation of yeast strains from makgeolli, tetracycline (10 μg/mL) and chloramphenicol (50 μg/mL) were added to YPD agar and PDA (0.4% potato starch, 2% dextrose, and 2% agar, Potato Dextrose Agar, BD Difco, USA). Yeast transformation was performed using the dimethyl sulfoxide method [14] with modifications. For selection of transformants, yeast cells were selected on synthetic complete plates composed of 0.67% yeast nitrogen base without amino acids (BD Difco) with 2% glucose and 0.77 g/L dropout supplementation with all required amino acids (Takara Bio, Japan) without uracil (SC-URA), or on YPD containing 100 μg/mL nourseothricin.

### Isolation and Identification of Makgeolli Yeasts by ITS Sequencing

**Fig. 1:**
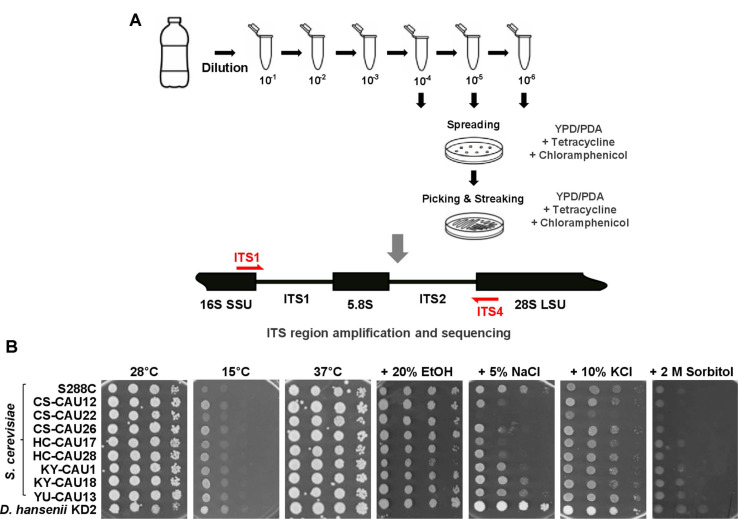
Isolation and growth phenotype analysis of yeast isolates from makgeolli. (**A**) Procedures for isolation and identification of yeasts by ITS sequencing. The primers ITS1 and ITS4, used for PCR amplification of rDNA fragment containing 5.8S, ITS1, and IT2 regions, are indicated by arrows. (**B**) Growth analysis of makgeolli yeast isolates under several stress conditions. Yeast cells were serially diluted from OD_600_ 1 to 0.1, 0.01, 0.001, spotted on YPD plates or YPD plates containing stress inducing components, and incubated for 3 days at 15°C, 28°C, or 37°C.

The commercially available makgeolli samples from 16 different local companies in Korea were serially diluted to 10^-4^, 10^-5^, and 10^-6^, whereupon 200 μL of each was applied to YPD or PDA plates with tetracycline and chloramphenicol (Fig. 1A). The genomic DNA (gDNA) was extracted from yeast strains by breaking yeast cells with glass beads in STES buffer (0.5 M NaCl, 0.2 M Tris-HCl [pH 7.5], 0.01M EDTA, 1% SDS) via vortexing for 3 minutes. Cell lysates were extracted by adding TE (pH 8.0) and phenol/chloroform/isoamyl alcohol (pH 8.0). After vortexing for 2 min and centrifugation of the cell lysate mixture, the supernatant containing gDNA was obtained and then subjected to precipitation by adding 0.3 M sodium acetate and 100% EtOH. DNA pellets were washed with 70% EtOH and dissolved in RNase water containing 25 μg/mL RNase A. Using the gDNA of each strain as template, PCR was performed using the primer set of ITS1 (TCC GTA GGT GAA CCT GCG G) and ITS4 (TCC TCC GCT TAT TGA TAT GC), and the obtained PCR products were subjected to sequencing (Fig. 1A).

### Ploidy Analysis, Whole Genome Sequencing, and Phylogenetic Tree Analysis

For ploidy analysis using flow cytometry, yeast cells inoculated at OD_600_ = 0.3 in YPD medium were cultivated and harvested at OD_600_ = 3. The cell pellet was fixed with absolute ethanol and resuspended in an RNase solution (2 mg/mL RNase A in 50 mM Tris-HCl, pH 7.5, and 15 mM NaCl) containing proteinase K. After washing, the cell pellet was finally resuspended in SYTOX solution (1 μM of SYTOX^TM^ green nucleic acid stain in 50 mM Tris, pH 7.5). Accuri™ C6 Plus flow cytometer (BD Biosciences, USA) and BD Accuri C6 software (BD Biosciences) were used for fluorescence-activated single-cell sorting analysis.

For whole-genome (WG) sequencing of *S. cerevisiae* strains, genomic DNA was extracted from spheroplasts and harvested by spooling as previously described [15]. Short-pair reads were generated using an Illumina NovaSeq 6000 sequencing system (Illumina, USA). Illumina reads were mapped into an erroneous draft assembly that generated a binary alignment map file, and the resulting alignment information was processed using Pilon (ver. 1.24) to obtain the final assembly. Phylogenetic tree analysis, initiated to classify *S. cerevisiae* at the strain level, was conducted using a gene set of the following strain-level classifications: YPR152C, YJL099W, YJL051W, YKL068W, YML056C, YNL161W, YNL125C, YAR042W, YBL052C, and YBR163W, as previously described [16].

### Construction of Functional *FDC1*-Overexpressing Makgeolli *S. cerevisiae* Strains

To introduce the *FDC1* expression vector YCpUT-ScFDC1-HA containing *URA3* as a selection marker [16] into the *fdc1*-deficient makgeolli *S. cerevisiae* strains, *ura3* auxotrophic mutants of the HC-CAU28 and YU-CAU13 strains were generated using CRISPR-Cas9 technology. The plasmid YCpNAT-C9U3g was constructed by using In-Fusion cloning (Takara Bio) to insert the *URA3* gRNA cassette from YCpH-C9U3g into a Cas9-NAT vector carrying the nourseothricin N-acetyltransferase (*NAT*) gene as a selection marker (Addgene, USA). To generate *ura3* auxotrophic mutants, the makgeolli strains were co-transformed with YCpNAT-C9U3g and donor *URA3* fragment containing a stop codon [17], and transformants were selected on a YPD plate containing 100 μg/mL nourseothricin (YPD+NAT). The resulting *ura3* strains, designated HC-CAU28-U3Δ and YU-CAU13-U3Δ, were subsequently transformed with YCpUT-ScFDC1-HA. Successful *URA*^+^ transformants were then selected on SC-URA plates. KY-CAU1 and KY-CAU18 were transformed with YCpNT-ScFDC1-HA [16], and transformants were selected on YPD+NAT plate.

### Volatile Flavor Analysis Using Headspace-Solid-Phase Microextraction with Gas Chromatography/Mass Spectrometry (HS-SPME GC/MS)

For flavor profiling of the makgeolli *S. cerevisiae* strains, yeast cells were inoculated at OD_600_ = 0.3 in 25 mL YPD medium and cultivated for 24, 48, and 72 h at 15°C or 28°C and at 220 rpm. To analyze the production of 4-VG, the yeast strains were cultivated in 25 mL YPD or SC-URA in the presence of 50 ppm ferulic acid for 24 h and 48 h [18]. The cell-free supernatant was obtained after centrifugation and transferred to a glass vial with a polytetrafluoroethylene/silicon septum (Agilent Technologies, USA) and subsequently analyzed using the splitless mode of the headspace-solid-phase microextraction with gas chromatography/mass spectrometry (HS-SPME GC/MS) system (7820A/5977E MSD, Agilent Technologies) with a DB-wax column (50 × 200 μm × 0.2 μm; Agilent Technologies), as described previously [19].

### Analysis of Transcript Levels of Volatile Flavor-Associated Genes in Makgeolli Yeast

Total RNA was extracted from yeast cells employing the method using glass beads combined with a purification kit, as previously described [19]. cDNA synthesis was performed according to the manufacturer's instructions using the ABScript Neo RT master mix (ABclonal Technology, USA). The relative transcript levels of volatile flavor biosynthetic genes were determined by qRT-PCR using TB Green Premix Ex Taq II (Takara Bio) and gene-specific primers in the CFX96 System (Bio-Rad, Hercules, USA). The expression levels of the target genes were normalized to *ACT1* as an endogenous control.

## Results

### Identification and Growth Characterization of Yeast Strains Isolated from Makgeolli

From 16 commercial makgeolli products in Korea [20], 28 yeast isolates (20 isolates from CS, 2 from KY, 1 from YU, and 5 from HC) were obtained (Table S2) and subjected to analysis of the 5.8S ribosomal RNA sequences flanking the internal transcribed spacer (ITS) regions 1 and 2 (Fig. 1A). Twenty ITS sequences of makgeolli isolates were identified that showed the highest identity with *S. cerevisiae* (> 99%) upon searching the BLAST ITS database; these sequences also matched *Saccharomyces cariocanus*, *Saccharomyces mikatae*, and *Saccharomyces paradoxus*, all of which belong to the *Saccharomyces sensu stricto* complex, with more than 98% identity (Table S2). Among these, 13 of the makgeolli isolates showed the second highest identity to *S. cariocanus*, whereas seven of them showed higher identity to *S. paradoxus* than to *S. cariocanus* (Table S2). *S. paradoxus* is exclusively isolated from natural sources, such as tree exudates, soil, and *Drosophila*; on rare occasions, it is isolated from fruit and fermented foods [21]. Recently classified within the *Saccharomyces sensu stricto* complex, *S. cariocanus* exhibits post-zygotic isolation from *S. paradoxus* [22]. To determine the taxonomic status of these closely related species, we performed a multiple sequence alignment of their ITS sequences. The alignment revealed that all the makgeolli isolates share identical ITS sequences with the *S. cerevisiae* strains derived from Japanese alcoholic beverages (awamori, sake, and shochu), while exhibiting minor nucleotide variations from the S288C reference and CBS1171 type strains (Fig. S1). This alignment clearly distinguished the makgeolli isolates from *S. cariocanus* and *S. paradoxus* at several specific polymorphic sites.

From the confirmed *S. cerevisiae* isolates, a subset of strains (CS-CAU12, CS-CAU22, CS-CAU26, HC-CAU17, HC-CAU28, KY-CAU1, KY-CAU18, and YU-CAU13) representing all four commercial makgeolli brands was initially selected to obtain information on the phenotypes of the makgeolli isolates. We analyzed their growth under different culture conditions (Fig. 1B). The results showed that the tested yeast isolates, except for CS-CAU22, grew better at a low temperature environment of 15°C when compared to the reference strain S288C, while no significant difference in the growth rate was detected at 28°C and 37°C (Fig. 1B). All the tested makgeolli isolates, together with the reference strain S288C, revealed high ethanol tolerance (up to 20%), in contrast to the completely inhibited growth of the 'Jang' yeast *Debaryomyces hansenii* KD2 [15] (Fig. 1B). To evaluate the osmotic tolerance of the makgeolli isolates, their growth on YPD plates containing KCl, NaCl, or sorbitol was examined. Under NaCl stress, strain CS-CAU22 showed almost no growth, whereas strains CS-CAU12, CS-CAU26, HC-CAU17, and HC-CAU28 showed less growth than the reference strain. In contrast, strains KY-CAU1, KY-CAU18, and YU-CAU13 grew similarly to the reference strain S288C. Unlike the growth under NaCl stress, all tested makgeolli isolates under KCl stress grew similarly to the reference strain S288C, except for CS-CAU22, which exhibited sensitivity to KCl stress. Regarding tolerance to sorbitol stress, strains CS-CAU12, CS-CAU22, CS-CAU26, HC-CAU17, and HC-CAU28 showed decreased or comparable growth, whereas strains KY-CAU1, KY-CAU18, and YU-CAU13 showed slightly better growth than the reference strain S288C. Notably, on YPD containing 5% (w/v) NaCl, 10% (w/v) KCl, and sorbitol, the 'Jang' yeast *D. hansenii* KD2 showed rigorous growth, consistent with a previous report which found it to be an osmotic-tolerant yeast [15].

### Evolutionary Genomic Features of Makgeolli Yeast Isolates

**Fig. 2:**
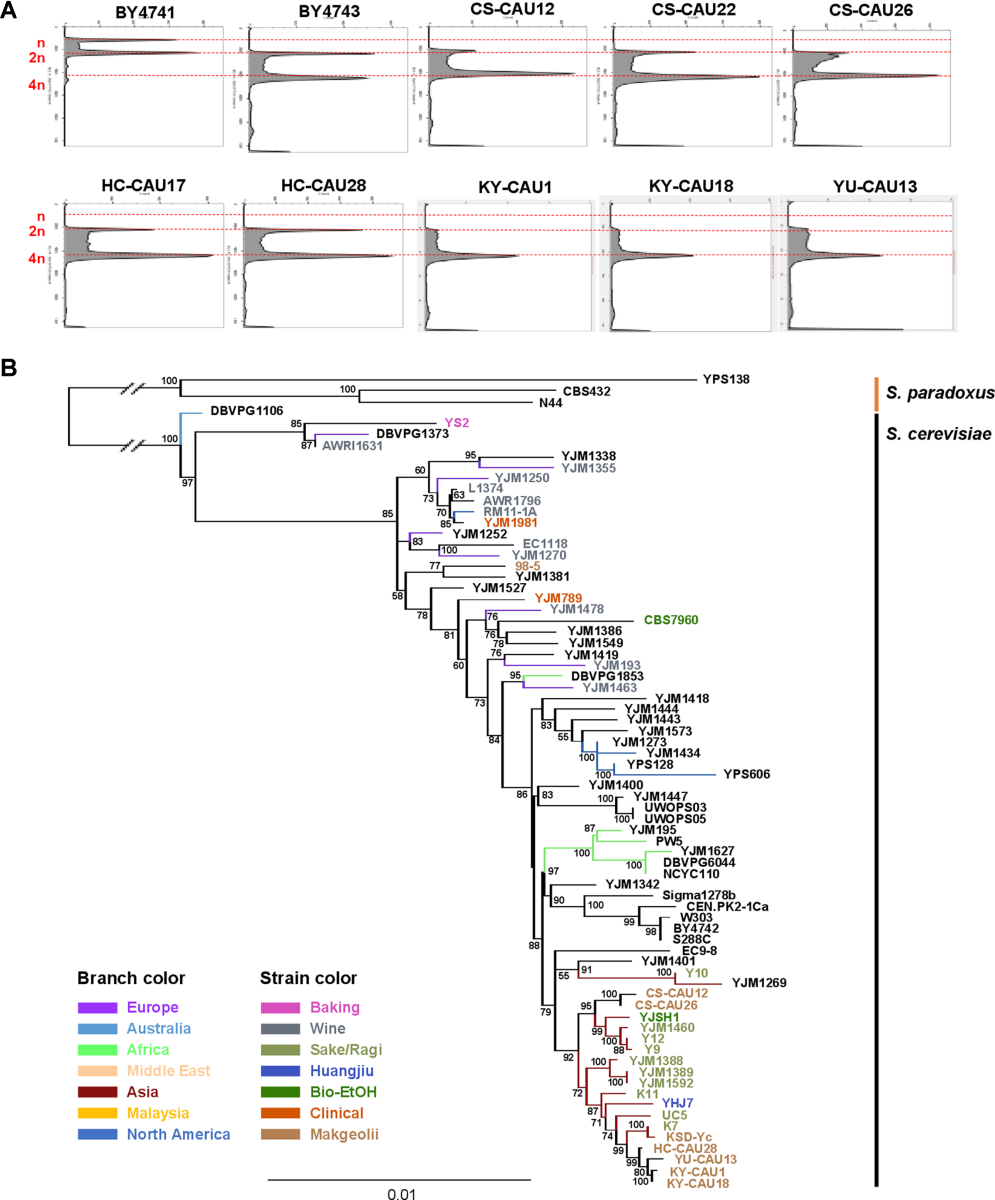
Ploidy and phylogenetic relationships of the makgeolli isolates. (**A**) Ploidy analysis of the yeast isolates from makgeolli. Nucleus of yeast cells fixed in absolute ethanol overnight were stained using SYTOX green and analyzed by flow cytometer. (**B**) Phylogenetic tree construction based on genome sequence information to classify *S. cerevisiae* at the strain level. The phylogenetic tree with maximum likelihood was constructed using the 10 concatenated *S. cerevisiae* genes—YPR152C, YJL099W, YJL051W, YKL068W, YML056C, YNL161W, YNL125C, YAR042W, YBL052C, and YBR163W—as previously described [16]. The bootstrap values greater than 50% are shown on the branches. The seven region types (Europe, Australia, Africa, Middle East, Asia, Malaysia, and North America) and seven source types (baking, wine, sake/ragi, huangjiu, bio-EtOH, clinical, and makgeolli) are represented as different colored branches and strains, respectively. Bar, 0.01 amino acid substitutions per site.

In an initial effort to examine the genomic features, the ploidy, the number of complete sets of chromosomes in a cell, of makgeolli yeast isolates was assessed using flow cytometry. Compared with the validated ploidy of *S. cerevisiae* BY4741 (haploid) and BY4743 (diploid), the ploidy of all makgeolli yeast isolates tested, including CS-CAU12, CS-CAU22, CS-CAU26, HC-CAU17, HC-CAU28, KY-CAU1, KY-CAU18, and YU-CAU13, was predicted to be diploid (Fig. 2A). To more accurately identify the makgeolli isolates at the genome level, six representative makgeolli yeast isolates, CS-CAU12, CS-CAU26, HC-CAU28, KY-CAU1, KY-CAU18, and YU-CAU13, were chosen based on their physiological robustness and subjected to WG sequencing analysis with Illumina short reads. The results of the WG sequencing are summarized in Table 2. Consistent with the result of the ploidy analysis, all six makgeolli yeast isolates have a diploid genome with sizes ranging from 23.10 Mb to 24.06 Mb. All six makgeolli isolates showed a GC rate of 38.1%.

Based on the WG sequencing data, a phylogenetic tree was reconstructed to include 71 *S. cerevisiae* strains representing diverse geographical locations and source backgrounds [16], along with three strains of *S. paradoxus* serving as an outgroup to root the tree (Fig. 2B). We used the concatenated nucleotide sequences of 10 genes from the S288C reference genome, which were considered phylogenetically informative [23], to infer the phylogenetic relationships. In the phylogenetic tree, all the analyzed makgeolli isolates clustered distinctly within the Asian-origin sake/ragi lineage of *S. cerevisiae*, suggesting that they share a common evolutionary ancestor adapted to traditional East Asian brewing environments, which typically involve rice-based fermentation. Interestingly, the makgeolli isolates diverged into two separate subclades. Strains HC-CAU28, YU-CAU13, KY-CAU1, and KY-CAU18 were closely grouped with the Japanese sake strain K7, which shares a common ancestor with the Chinese rice wine strain YHJ7 [24]. In contrast, the CS-CAU12 and CS-CAU26 strains were positioned close to the Chinese industrial bioethanol strain YJSH1 [24] and the Indonesian ragi strain Y9 [25]. This intra-lineage divergence implies that although makgeolli yeasts originated from a shared Asian fermentation ancestor, they have undergone distinct evolutionary trajectories or localized domestication events.

### Comparison of Volatile Flavor Profiles of Makgeolli isolates

**Fig. 3:**
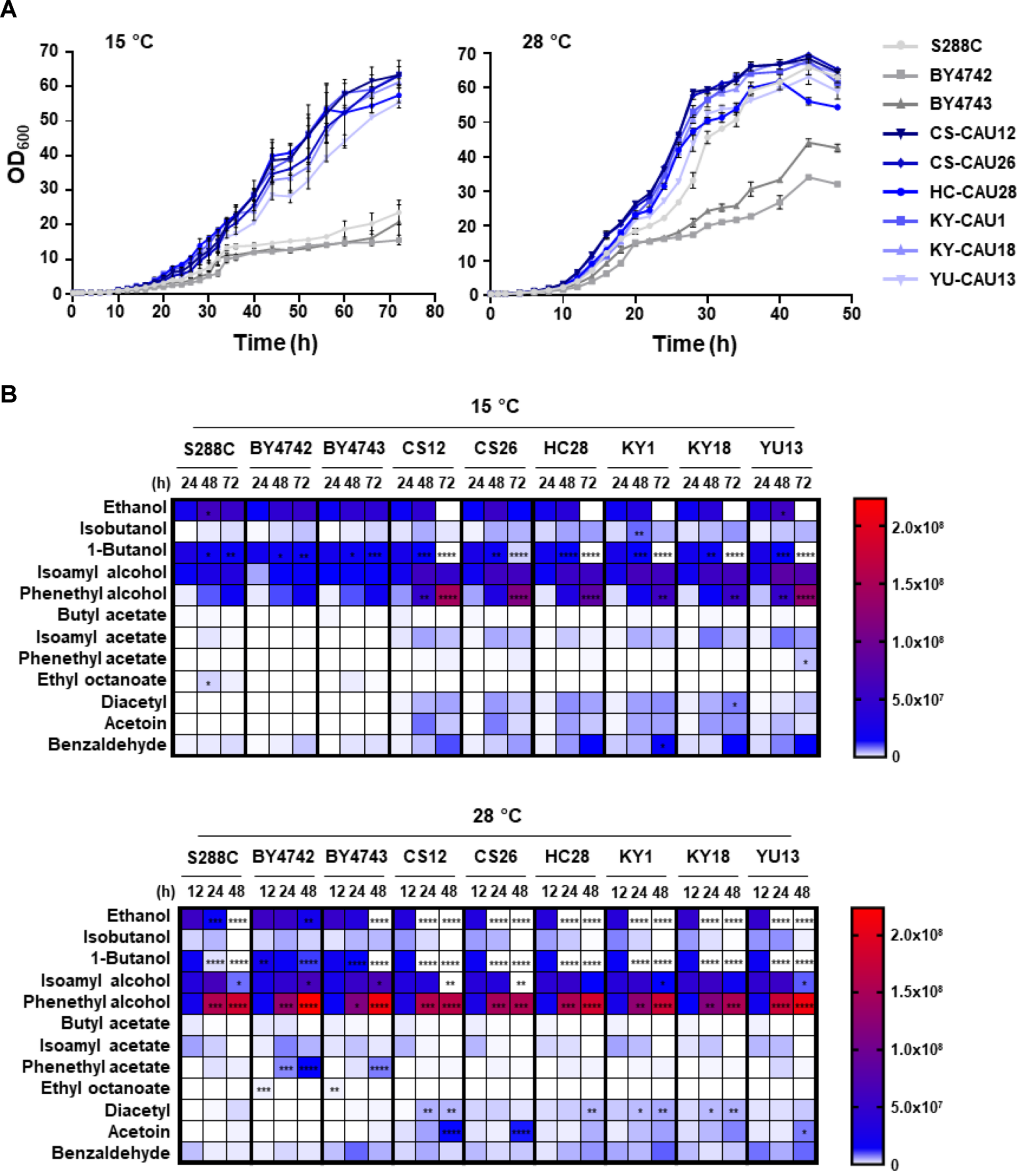
Growth and volatile flavors of YPD-cultivated makgeolli isolates at 15°C and 28°C. (**A**) Growth curves of the following yeast strains: S288C, BY4742, BY4743, CS-CAU12, CS-CAU26, HC-CAU28, KY-CAU1, KY-CAU18, and YU-CAU13. Data are presented as the mean and standard error of the mean from biological duplicate experiments. (**B**) Heatmap of volatile flavor components produced by the S288C reference strain, two lab strains (BY4742 and BY4743), and the makgeolli isolates including CS-CAU12 (CS12), CS-CAU26 (CS26), HC-CAU28 (HC28), KY-CAU1 (KY1), KY-CAU18 (KY18), and YU-CAU13 (YU13) at 15°C and 28°C analyzed by HS-SPME GC/MS. The analysis was performed in duplicate. GraphPad Prism (ver. 8.4) was used to generate the plots. *, *p* < 0.05; **, *p* < 0.01; ***, *p* < 0.001; and ****, *p* < 0.0001, between cell cultures and culture of S288C at 24 h (15°C) or at 12 h (28°C) (one-way ANOVA).

Several previous studies have reported that wines produced at low temperatures (10–15°C) generated wines with more aromatics, although this effect was strain-dependent [26, 27]. The *S. cerevisiae* makgeolli isolates exhibited tolerance to low temperatures in the spotting analysis (Fig. 1B). To determine whether cold tolerance is either a specific characteristic of the makgeolli isolates or a consequence of ploidy (*i.e.*, diploidy), we compared the growth curves of the makgeolli isolates with those of the *S. cerevisiae* S288C reference strain and two *S. cerevisiae* laboratory strains, BY4742 (haploid) and BY4743 (diploid), cultivated in YPD medium at 15°C and 28°C (Fig. 3A). All six makgeolli isolates grew faster than the reference strain and the lab strains at both temperatures, regardless of ploidy status, and their robust growth was much more evident at 15°C.

We compared the volatile flavor profiles of the makgeolli isolates with those of the *S. cerevisiae* S288C reference strain and the two lab strains cultivated in YPD medium at 15°C and 28°C. A heatmap based on the HS-SPME GC-MS analysis showed that more diverse volatile flavor compounds were produced from the makgeolli isolates, especially at 15°C, and they were retained for a longer duration at low temperature than at 28°C (Fig. 3B). In the makgeolli isolates, the level of ethanol increased up to 48 h but decreased later at 15°C, mainly due to the consumption of ethanol as a carbon source after the depletion of glucose. The amount of ethanol was almost undetectable after 24 h at 28°C in makgeolli isolates, suggesting rapid metabolic consumption due to their robust growth at this temperature (Fig. 3B). At 15°C, the makgeolli isolates notably generated higher levels of diacetyl and acetoin, along with slight increases in isoamyl acetate, isoamyl alcohol, phenethyl alcohol, and benzaldehyde, compared to the reference and lab strains. Interestingly, the enhanced production of benzaldehyde in the makgeolli isolates was highly specific to low-temperature conditions. At 28°C, the overall profile shifted significantly. While the production of diacetyl and acetoin was still remarkable in the makgeolli isolates, other volatile flavor compounds such as isobutanol and isoamyl acetate diminished after 24 h. Furthermore, the levels of benzaldehyde became negligible and indistinguishable from those of the reference and lab strains. Unlike other volatiles, phenethyl alcohol accumulated to very high levels at 28°C across all the strains. This indicates that while fermentation at low temperatures generally favors aroma complexity and balance by retaining volatile esters and specific aldehydes in fermented beverages and foods [28, 29], higher temperatures may enhance the production of specific fusel alcohols, such as phenethyl alcohol, via the Ehrlich pathway [26].

### Expression Analysis of Genes Involved in the Formation of Volatile Flavors in Makgeolli Isolates

**Fig. 4:**
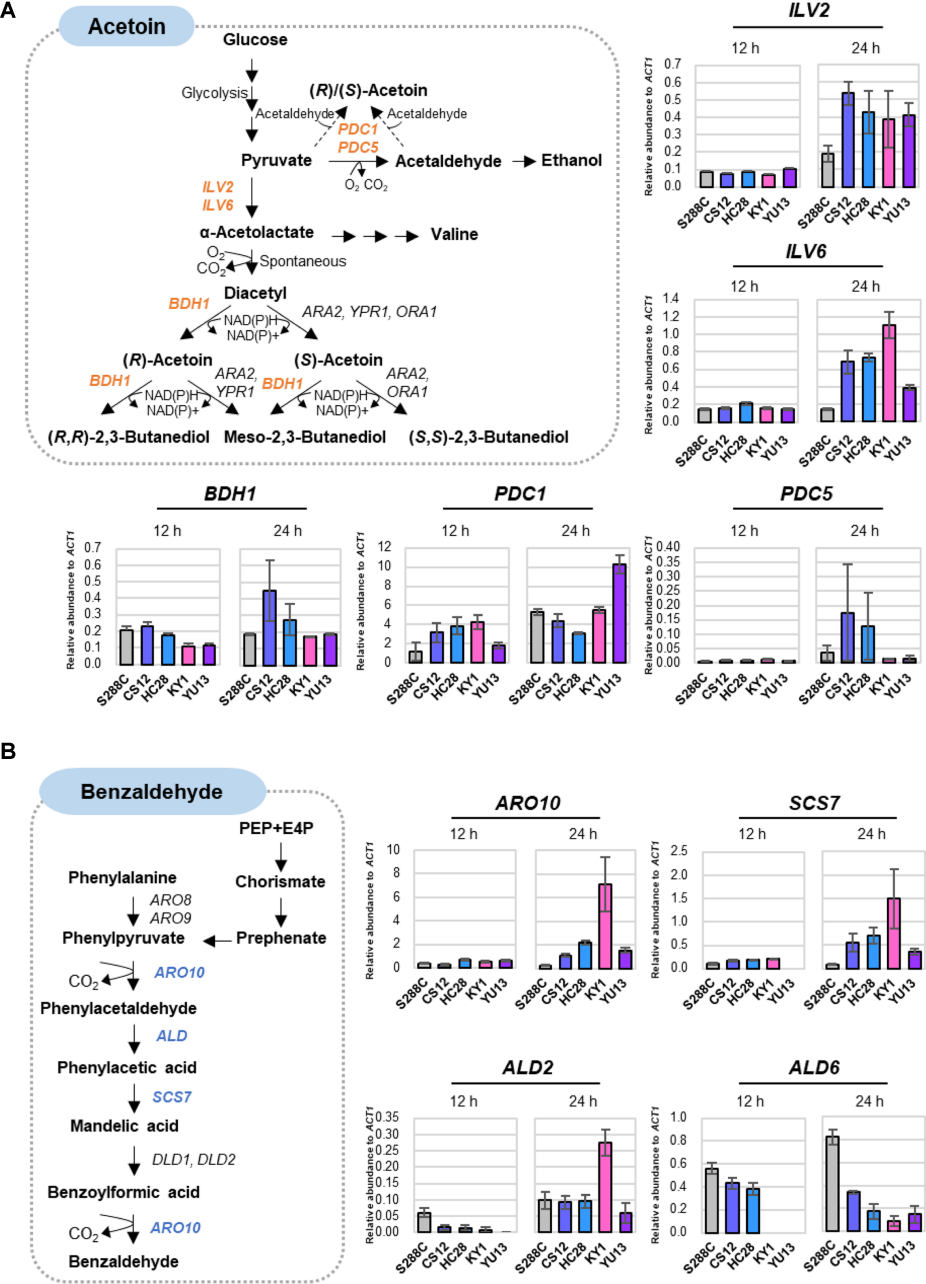
qRT-PCR analysis of acetoin and benzaldehyde biosynthesis genes in makgeolli isolates. (**A**) Biosynthetic pathway of acetoin from pyruvate and the relative expression levels of the corresponding genes. (**B**) Biosynthetic pathway of benzaldehyde from phenylalanine and chorismate and the relative expression levels of the corresponding genes. The analyzed genes were marked in bold font and color. The transcript levels at 12 h and 24 h are shown as relative ratios to those of the *ACT1* gene. Error bars represent standard error of the mean from biological duplicate measurements.

To determine whether the higher production of diacetyl, acetoin, and benzaldehyde detected in the makgeolli isolates was due to the increased expression of their corresponding biosynthesis genes, we performed qRT-PCR analysis to examine their transcript levels in the makgeolli isolates cultivated in YPD at 28°C (Fig. 4). *S. cerevisiae* naturally produces α-acetolactate synthase, encoded by *ILV2* and *ILV6*. Under aerobic conditions, spontaneous decarboxylation of (*S*)-α-acetolactate leads to the formation of diacetyl, which is subsequently converted into (*R*)- or (*S*)-acetoin by endogenous reductases (Fig. 4A) [30]. Additionally, acetoin can be generated through side reactions with pyruvate decarboxylases (PDCs) via the condensation of pyruvate, acetaldehyde, or two acetaldehyde molecules (Fig. 4A) [30]. Acetoin can be further reduced to (*R,R*)-, meso-, or (*S,S*)-2,3-butanediol by (*R*)- or (*S*)-alcohol-forming reductases. The mRNA levels of *ILV2* and *ILV6* were generally higher in the makgeolli isolates at 24 h than in the S288C reference strain (Fig. 4A). In contrast, the expression levels of *BDH1*, *PDC1*, and *PDC5* did not exhibit any obvious pattern across the makgeolli isolates compared to the reference strain. These results suggest that the enhanced accumulation of diacetyl and acetoin observed in the makgeolli isolates is primarily driven by active metabolic flux through the α-acetolactate pathway, facilitated by the increased transcription of *ILV2* and *ILV6*, rather than through *PDC*-mediated side reactions.

Benzaldehyde is synthesized from sugars either through the chorismate pathway or via the aromatic amino acid phenylalanine [31]. In this pathway, the intermediate phenylpyruvate is decarboxylated to phenylacetaldehyde by *ARO10*, which encodes phenylpyruvate decarboxylase. Benzoylformic acid, formed in three steps via the action of *ALD* (aldehyde dehydrogenases), *SCS7* (sphingolipid alpha-hydroxylase) and *DLD1/2* (D-lactate dehydrogenases), is subsequently converted to benzaldehyde by *ARO10* (Fig. 4B). Consistent with the elevated benzaldehyde production observed in the makgeolli isolates at 15 °C (Fig. 3B), qRT-PCR analysis revealed a significant upregulation of these key biosynthesis genes compared to the reference strain (Fig. 4B). At 24 h, the transcript levels of *ARO10* and *SCS7* were more than two-fold higher in the makgeolli isolates than in S288C. Notably, this upregulation was pronounced in KY-CAU1, corroborating its significantly higher benzaldehyde accumulation (Fig. 3B). Furthermore, the expression of *ALD2*, which encodes an aldehyde dehydrogenase responsible for providing the intermediate phenylacetic acid in this pathway, was also exceptionally elevated in KY-CAU1, whereas the expression of *ALD6* was substantially suppressed in all makgeolli isolates compared to S288C. This synchronized upregulation of *ARO10*, *ALD2*, and *SCS7* suggests that KY-CAU1 possesses a highly active metabolic flux directed toward benzaldehyde biosynthesis.

### Comparison of 4-VG Production and Biosynthetic Genes of Makgeolli Yeasts

**Fig. 5:**
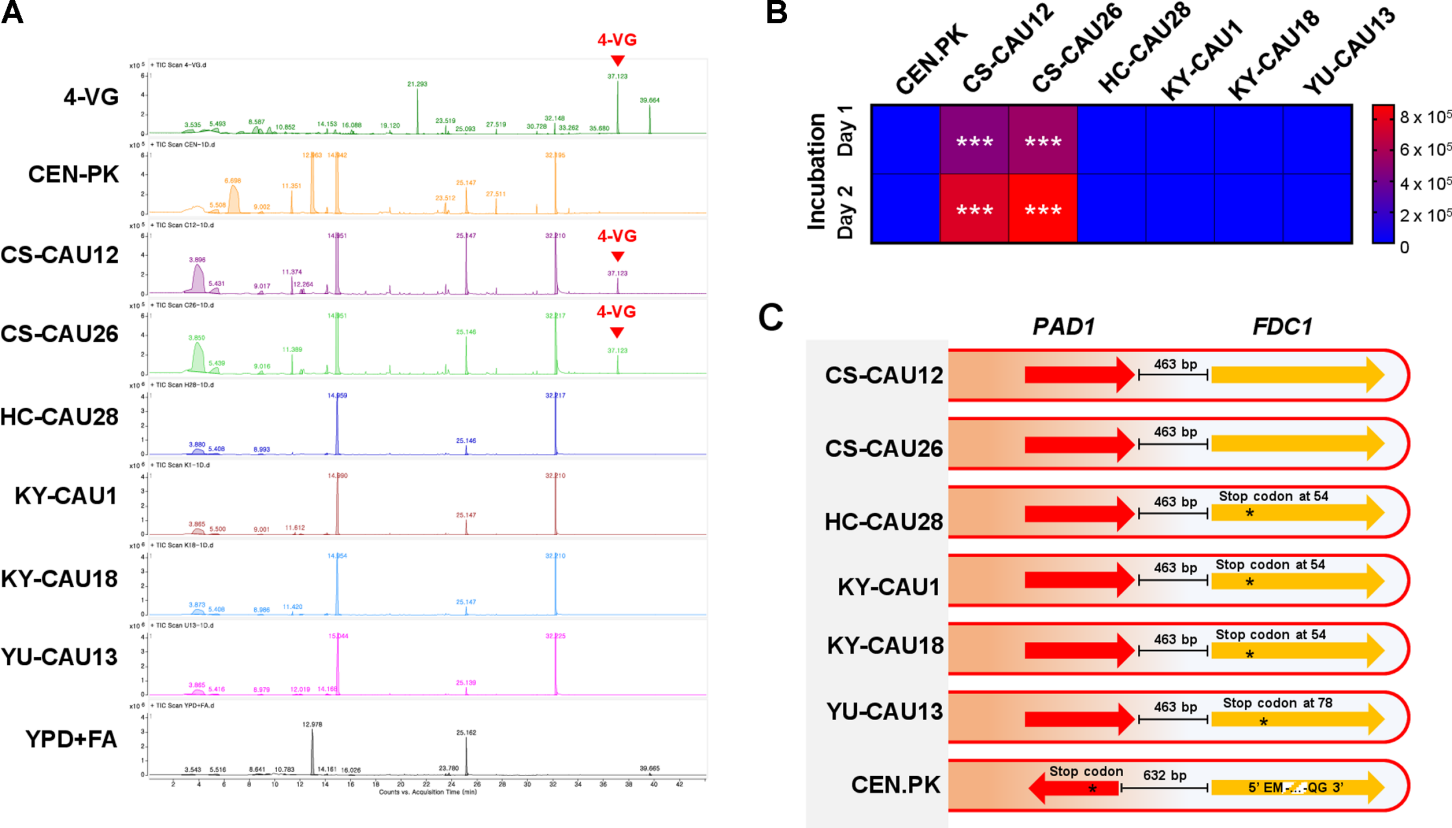
Comparative analysis of the 4-vinylguaiacol (4-VG) bioconversion activity of the makgeolli isolates. Yeast cells were grown in YPD containing 50 ppm ferulic acid, and samples were collected after 1 and 2 days of incubation. (**A**) 4-VG profiles of *S. cerevisiae* CEN.PK2-1C, CS-CAU12, CS-CAU26, HC-CAU28, KY-CAU1, KY-CAU18 and YU-CAU13 strains analyzed by HS-SPME GS/MS. (**B**) Heatmap of 4-VG production in the makgeolli isolates. (**C**) Schematic representation of the *PAD1* and *FDC1* genes required for 4-VG bioconversion in the *S. cerevisiae* strains. The red lines represent chromosomes, indicating the subtelomeric location of the *PAD1* and *FDC1* genes on the right arm of chromosome IV.

The presence of 4-VG has been reported in some makgeolli products [32, 33]. 4-VG, a phenolic compound with a smoke-like flavor, is produced by yeast via the decarboxylation of ferulic acid, an abundant phenolic compound found in plant cell walls. The decarboxylation of ferulic acid in yeast is mostly mediated by the *FDC1*-encoded decarboxylase, which requires a flavin-derived cofactor encoded by *PAD1* [34]. We examined the 4-VG production ability of makgeolli isolates cultured in YPD containing 50 ppm ferulic acid by analyzing the bioconversion of ferulic acid to 4-VG. Loss of 4-VG production was observed in the CEN.PK, HC-CAU28, KY-CAU1, KY-CAU18, and YU-CAU13 strains, while the 4-VG production phenotype (4-VG^+^) was observed in the CS-CAU12 and CS-CAU26 strains (Fig. 5A and 5B).

Comparison of the *PAD1* and *FDC1* gene sequences, which are clustered in the subtelomeric region of the right arm of chromosome IV in the *S. cerevisiae* strains [16], revealed notable nonsense mutations in the *FDC1* gene of HC-CAU28 (stop codon at the 54^th^ position), KY-CAU1 (stop codon at the 54^th^ position), KY-CAU18 (stop codon at the 54^th^ position), and YU-CAU13 (stop codon at the 78^th^ position) strains, while there were no mutations in the *PAD1* gene (Figs. 5C and Fig. S2). To determine whether these mutations are homozygous or heterozygous in the diploid isolates, we examined thoroughly the nucleotide sequence of *FDC1* from the WG data (Fig. S3A) and noticed that the strain KY-CAU1 has heterozygous *FDC1* alleles. Sanger sequencing of the PCR-amplified *FDC1* genes confirmed that KY-CAU1 possesses the heterozygous *FDC1* alleles carrying the wild-type and the nonsense mutant alleles, while the HC-CAU28, KY-CAU18, and YU-CAU13 strains have homozygous *FDC1* alleles with the nonsense mutations, leading to premature truncation of *FDC1* protein (Fig. S3B). This KY-CAU1-specific heterozygosity was also found in other genetic loci, such as *KRE28* and *RBA50* (data not shown), indicating that the genome of KY-CAU1 is heterozygous diploid. The presence of a premature stop codon mutation detected in the *FDC1* gene of these strains may be the main contributor to the loss of 4-VG production. In contrast, the CS-CAU12 and CS-CAU26 strains, which produced considerable amounts of 4-VG, retained both wild-type *PAD1* and *FDC1* (Figs. 5, S2, and Fig. S3). In the case of the *S. cerevisiae* strain CEN.PK2-1C, which lacks 4-VG production activity, it has a stop codon mutation at the 98th amino acid position in *PAD1* along with additional alterations, including an extended space length between the *PAD1* and *FDC1* genes, an inverted orientation of *PAD1*, and deletion of *FDC1* corresponding to the 265–295^th^ amino acids [16]. These results support a positive relationship between the sequence variation of *PAD1* and/or *FDC1* and the ferulic acid decarboxylation ability of various yeast strains. To explicitly present the genotype-phenotype relationship, we summarized the *FDC1* mutations and their zygosity in a strain-by-strain manner (Table 3).

**Fig. 6:**
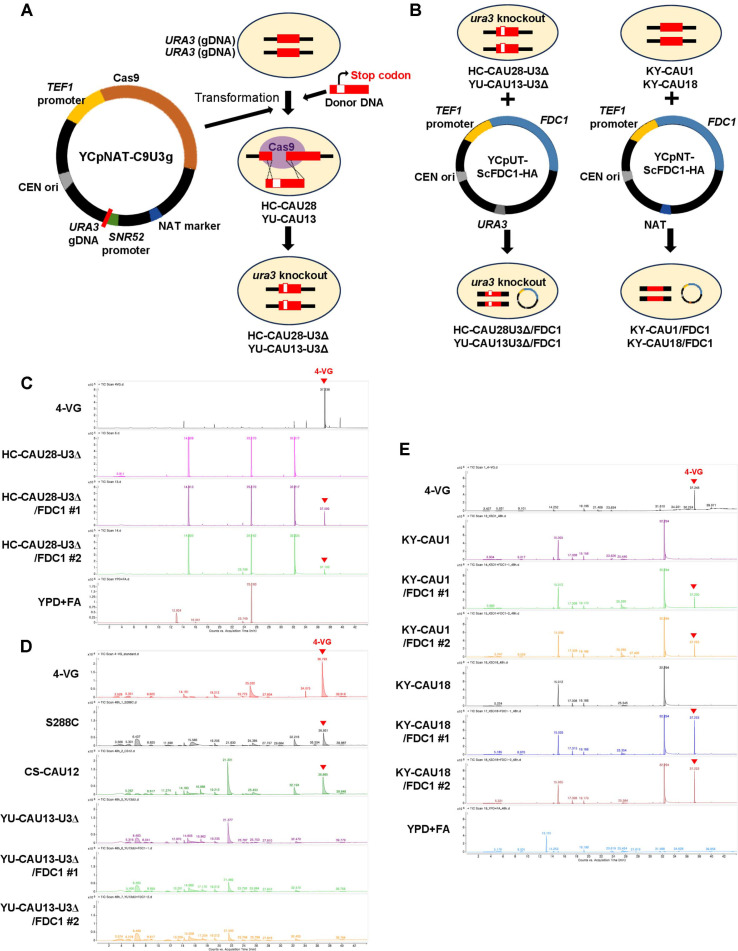
Recovery of 4-VG formation of the makgeolli isolates by *FDC1*-complementation. (**A**) Scheme of the generation of *ura3*^-^ auxotroph of the makgeolli isolates, HC-CAU28 and YU-CAU13, using CRISPR/Cas9, resulting in HC-CAU28-U3Δ and YU-CAU13-U3Δ, respectively. (**B**) Transformation with the *FDC1* expression vectors. The HC-CAU28-U3Δ and YU-CAU13-U3Δ were transformed with YCpUT-ScFDC1-HA, and KY-CAU1 and KY-CAU18 with YCpNT-ScFDC1-HA. (**C-E**) 4-VG production of the *fdc1*-makgeolli strains by introduction of wild-type *FDC1*. (**C**) Chromatograms of HC-CAU28-U3Δ and its *FDC1* transformants cultivated in YPD medium containing 50 ppm ferulic acid. (**D**) Chromatograms of YU-CAU13-U3Δ and its *FDC1* transformants cultivated in SC-URA minimal medium containing 50 ppm ferulic acid. S288C and CS-CAU12 were included as positive controls for 4-VG production. (**E**) Chromatograms of KY-CAU1 and KY-CAU18 and their *FDC1* transformants cultivated in YPD medium containing 50 ppm ferulic acid. Samples were analyzed after 2 day-incubation through HS-SPME GC/MS.

To determine whether the ability to produce 4-VG was restored after introducing wild-type *FDC1* by transformation with the episomal vector using *URA3* as the selection marker, a *ura3* mutant strain was generated using the CRISPR/Cas9 system. After transformation of strains HC-CAU28 and YU-CAU13 with the YCpNAT-C9U3g vector in the presence of donor *URA3* DNA fragment containing a stop codon mutation [17], transformants selected on YPD containing nourseothricin were screened for Ura- phenotype by confirming the absence of their growth on SC-URA (Fig. 6A). The obtained *ura3*^-^ strains, HC-CAU28-U3Δ and YU-CAU13-U3Δ (Table 1), were subsequently transformed with the YCpUT-ScFDC1-HA vector expressing the functional *FDC1* under control of the *TEF1* promoter and GAL7 terminator (Fig. 6B, Table S1). This restored 4-VG production in HC-CAU28-U3Δ, generating the active transformants HC-CAU28UΔ/FDC1 (Fig. 6C). On the other hand, we could not observe the restoration of 4-VG production ability in YU-CAU13-U3Δ, even in cultivation with a selective SC-URA medium to maintain plasmid stability (Fig. 6D). In the case of strains KY-CAU1 and KY-CAU18, in which the application of CRISPR-Cas9 to the *ura3*^-^ strain generation was unsuccessful, the *FDC1* overexpression cassette was introduced into the strains using YCpNT-ScFDC1-HA carrying NAT as a selection marker. The introduction of *FDC1* also restored the 4-VG production ability of KY-CAU1 and KY-CAU18 (Fig. 6B and 6E). It is notable that the extent of restoration by *FDC1* complementation varied significantly among the strains. While *FDC1* complementation resulted in robust 4-VG production in KY-CAU18, it led to only partial or weak restoration in HC-CAU28 and KY-CAU1, and no restoration in YU-CAU13. These results indicate that the functional expression of *FDC1* alone was insufficient to fully restore 4-VG production in certain genetic backgrounds.

## Discussion

Given the increasing worldwide popularity of the traditional Korean rice wine makgeolli [35, 36], research on the identification and functional characterization of the key microorganisms in makgeolli brewing has attracted increasing attention. While the traditional method for makgeolli brewing relies solely on the microorganisms present in nuruk, commercial makgeolli production is frequently supplemented with an additional inoculation of *S. cerevisiae*. In the present study, we identified and characterized yeast isolates from commercial makgeolli products in South Korea. All makgeolli isolates were identified as *S. cerevisiae* with diploid genomes (Fig. 2A), a characteristic often observed in the genomes of various yeast species isolated from fermented foods. Our previous studies on the comparative genomics of yeast species isolated from traditional Korean fermentation products, such as nuruk and ganjang, have revealed the frequent presence of heterozygous diploid genomes as an evolutionary strategy in the fermentation environment [4, 16, 37]. A previous study analyzing human-related *S. cerevisiae* subpopulations reported that wine and sake isolates are primarily diploids and monophyletic with limited genetic diversity, whereas *S. cerevisiae* beer isolates are polyphyletic and characterized by higher ploidy (≥ 3*n*) with a higher number of aneuploidy events [38].

Phylogenetic analysis, based on the genomic sequences of more than 70 *S. cerevisiae* strains representing diverse geographical locations and source backgrounds, classified the makgeolli isolates within the Asian sake/ragi lineage (Fig. 2B). Their close clustering with the Japanese sake (K7) and Chinese Huangjiu (YHJ7) strains strongly suggested a shared domestication trajectory. Unlike Western wine or ale yeasts, these East Asian strains have co-evolved with a unique “simultaneous saccharification and fermentation” process using rice-based substrates, adapting to specific environmental pressures [24, 39]. This shared evolutionary background likely provides the fundamental genetic basis for their cold tolerance at 15°C and superior volatile flavor profiles compared to the reference strain [40]. Interestingly, our phylogenetic tree also revealed distinct intra-lineage divergence among the makgeolli isolates, implying localized adaptation or varying selection criteria. The subclade comprising HC-CAU28, KY-CAU1, KY-CAU18, and YU-CAU13 clustered tightly with traditional sake and rice wine strains, whereas the CS-CAU12 and CS-CAU26 strains showed a closer phylogenetic relationship with the Chinese industrial bioethanol strain YJSH1, indicating evolutionary optimization geared toward flavor complexity and balanced fermentation [40].

The close relationship between the phylogenetic position based on genomic information and metabolic traits was further validated by comparing the makgeolli yeast strains isolated in this study with other previously reported strains, KSD-YC and 98-5, used for commercial makgeolli production (Fig. S4A) [16]. The flavor profiles of the makgeolli strains isolated in this study were highly comparable to those of KSD-YC, which is consistent with their close genetic relationships in the phylogenetic tree (Fig. 2B). In contrast, 98-5 displayed a slightly different flavor profile; for example, lower amounts of key volatile compounds such as phenethyl alcohol and phenethyl acetate reflected its distinct phylogenetic position (Fig. S4A, Fig. 2B). Collectively, these findings emphasize that these makgeolli isolates have undergone domestication in specific brewing environments, making them suitable for producing alcoholic beverages with different flavors.

Diacetyl is known for its strong, intense butter or butterscotch taste with a very low flavor threshold. The presence of diacetyl above its threshold in beer is generally considered an off-flavor, except in specific beer styles such as English and Czech Pilsners [41]. Unlike diacetyl, acetoin contributes to a mild, neutral buttery or creamy aroma with a much higher flavor threshold than diacetyl, making it less of an off-flavor issue and therefore acceptable at typical concentrations [42]. Although the makgeolli isolates exhibited increased diacetyl production compared to the reference strain, their accumulation of acetoin was substantially higher, particularly in a temperature-dependent manner (Fig. 3B, Fig. S4A).

Interestingly, under YPD cultivation, the makgeolli isolates showed decreased expression levels of *ALD6*, the major cytosolic aldehyde dehydrogenase [43], compared to the reference strain (Fig. 4B). By downregulating this primary acetate-producing enzyme, makgeolli yeasts may effectively prevent the excessive accumulation of acetic acid, thereby avoiding strong sourness [44]. Notably, KY-CAU1 showed higher expression levels of *ARO10*, *SCS7*, and *ALD2* involved in the benzenoid pathway to channel metabolic intermediates toward benzaldehyde biosynthesis than the other yeast isolates. This may indicate specialized domestication traits for brewing KY makgeolli products with a bitter taste. Although the overall accumulation of volatile esters in the makgeolli isolates did not significantly exceed that in the reference strain, qRT-PCR analysis revealed highly dynamic ester turnover (Fig. S4). The makgeolli isolates exhibited upregulated transcription of ester-synthesizing genes, such as *ATF1*, *EEB1*, *EHT1*, and *EAT1*, along with the concomitant upregulation of *IAH1*, an ester-degrading esterase, at 24 h (Fig. S4B) [45]. This co-upregulation suggested a rapid dynamic equilibrium between ester synthesis and hydrolysis. Therefore, the ester turnover cycle might serve as a crucial adaptive mechanism in makgeolli yeasts to prevent ester over-accumulation, thereby maintaining a refined and balanced fruity aroma. However, the expression of the *PAD1* and *FDC1* genes at the transcriptional level did not directly reflect the 4-VG production of the strains analyzed, and the lack of 4-VG production capacity was mainly due to mutations in the biosynthetic genes (Fig. S4B). The metabolic profiles and gene expression patterns during actual makgeolli brewing may be substantially different from those observed under laboratory culture conditions because of the complex matrix of rice substrates and the diverse microbial consortia present in nuruk. Nevertheless, the results of this study provide information on the inherent capacity and basal level of gene expression associated with flavor production.

With increasing WG sequencing of yeast species that produce flavor components in foods [46], genome-based exploration of volatile flavor diversity in food yeast species would facilitate the identification of target genes to be modulated for optimized flavor profiles. For example, the production of 4-VG varies among yeast isolates. While some strains retained their 4-VG production ability, others exhibited loss-of-function mutations in *PAD1* and/or *FDC1*, highlighting the impact of genetic variations on flavor profiles and yeast domestication in the brewing environment [11]. Our sequence analysis based on the WG sequencing revealed that the 4-VG-negative phenotype of the makgeolli isolates (HC-CAU28, KY-CAU1, KY-CAU18, and YU-CAU13) is strongly associated with the presence of a premature stop codon in the *FDC1* gene (Fig. 5C, Fig. S2, and Fig. S3). To validate whether nonsense mutations are key genetic factors contributing to the phenotype, we performed functional complementation. However, the lack of stable auxotrophic markers in industrial yeast strains poses a significant hurdle to genetic manipulation. To overcome this, we successfully employed CRISPR/Cas9-mediated genome editing to engineer an *ura3* auxotrophic background in the diploid HC-CAU28 and YU-CAU13 isolates. Interestingly, the CRISPR/Cas9 system was not universally effective across all makgeolli isolates, such as KY-CAU1 and KY-CAU18, reflecting strain-specific differences in inherent genomic and physiological features among industrial yeasts (Fig. 6). Intriguingly, while the introduction of wild-type *FDC1* robustly restored 4-VG production in KY-CAU18, it resulted in only partial recovery in HC-CAU28 and KY-CAU1 and failed in YU-CAU13. This varying degree of complementation indicates that the nonsense mutation in *FDC1* is a key determinant, but not the sole genetic factor controlling the 4-VG-negative phenotype. Additional genetic defects, such as differences in precursor availability due to transporter efficiency, deficient cofactor supply, or other unknown interactions within their diverse genetic backgrounds, likely co-determine the final flavor profile in these industrial strains. Moreover, under our culture conditions (12 h and 24 h), the transcript levels of *FDC1* were relatively low and did not show significant induction upon prolonged exposure to ferulic acid (Fig. S5). This low basal transcript abundance strongly correlates with the previously reported Fdc1/Act1 protein abundance ratio (~5%) in the *Saccharomyces* Genome Database (https://www.yeastgenome.org/). Consistent with the indistinct expression patterns cataloged in the database, specific environmental cues or transcriptional regulations governing *FDC1* expression have not yet been reported. Thus, systematic investigation should be performed to elucidate the regulatory mechanisms of *FDC1* in these industrial isolates.

Overall, the present study provides valuable insights into the genomic features and flavor-producing capacity of *S. cerevisiae* strains used for induced fermentation in brewing commercial makgeolli products, along with genetic manipulation platforms. These findings will facilitate the rational design of yeast starters tailored to produce rice wine with specific flavor profiles and enhanced nutraceutical compositions.

## Supplemental Materials

Supplementary data for this paper are available on-line only at http://jmb.or.kr.



## Figures and Tables

**Table 1:** Yeast strains used in this study.

Strain	Genotype / Origin (Makgeolli)	Reference
***Saccharomyces cerevisiae***		
S288C	*MATα SUC2 gal2 mal2 mel flo1 flo8-1 hap1 ho bio1 bio6*	ATCC 204508
BY4741	*MATa his3Δ1 leu2Δ0 met15Δ0 *ura3*Δ0*	Open Biosystems
BY4742	*MATα his3Δ1 leu2Δ0 lys2Δ0 *ura3*Δ0*	Open Biosystems
BY4743	*MATa/MATα his3Δ1/his3Δ1 leu2Δ0/leu2Δ0 lys2Δ0/+ met15Δ0/+ *ura3*Δ0/*ura3*Δ0*	Open Biosystems
CEN.PK2-1C	*MATa *ura3*-52 leu2-3_112 trp1-289 his3Δ1 MAL2-8c SUC2*	EUROSCARF:30000A
CS-CAU12	WT isolated from CS makgeolli	This study
CS-CAU22	WT isolated from CS makgeolli	This study
CS-CAU26	WT isolated from CS makgeolli	This study
HC-CAU17	WT isolated from HC makgeolli	This study
HC-CAU28	WT isolated from HC makgeolli	This study
HC-CAU28-U3Δ	*ura3* auxotroph of HC-CAU28	This study
HC-CAU28-U3Δ/FDC1 #1	HC-CAU28-U3Δ harboring YCpUT-*ScFDC1*-HA	This study
HC-CAU28-U3Δ/FDC1 #2	HC-CAU28-U3Δ harboring YCpUT-*ScFDC1*-HA	This study
KY-CAU1	WT isolated from KY makgeolli	This study
KY-CAU1/FDC1 #1	KY-CAU1 harboring YCpNT-*ScFDC1*-HA	This study
KY-CAU1/FDC1 #2	KY-CAU1 harboring YCpNT-*ScFDC1*-HA	This study
KY-CAU18	WT isolated from KY makgeolli	This study
KY-CAU18/FDC1 #1	KY-CAU18 harboring YCpNT-*ScFDC1*-HA	This study
KY-CAU18/FDC1 #2	KY-CAU18 harboring YCpNT-*ScFDC1*-HA	This study
YU-CAU13	WT isolated from YU makgeolli	This study
YU-CAU13-U3Δ	*ura3* auxotroph of YU-CAU13	This study
YU-CAU13-U3Δ/FDC1 #1	YU-CAU13-U3Δ harboring YCpUT-*ScFDC1*-HA	This study
YU-CAU13-U3Δ/FDC1 #2	YU-CAU13-U3Δ harboring YCpUT-*ScFDC1*-HA	This study
KSD-YC	KSD strain for makgeolli production	[16]
98-5	KFRI strain for makgeolli production	[16]
***Debaryomyces hansenii***		
KD2	WT isolated from ganjang	[15]

**Table 2:** Whole genome sequencing summary of makgeolli yeast isolates.

*S. cerevisiae* strains	No. of scaffolds	N50 (Kb)	L50	GC (%)	BUSCO completeness (%)	Genome size (Mb)	NCBI Accession Number
S288C	17	924.4	6	38.2	99.4	12.2	GCA_000146045.2
CS-CAU12	252	906.6	6	38.1	98.3	24.06	SRR36368407
CS-CAU26	255	900.7	6	38.1	98.4	23.96	SRR36368406
HC-CAU28	114	892.3	6	38.1	99.6	23.12	SRR36368405
KY-CAU1	75	891.2	6	38.1	99.7	23.10	SRR36368404
KY-CAU18	85	889.9	6	38.1	99.7	23.12	SRR36368403
YU-CAU13	78	891.7	6	38.1	99.5	23.12	SRR36368402

Cheongju Sinseonju (CS) makgeolli, Haechang (HC) raw makgeolli, Kuksundang Yetnal (KY) makgeolli, and Yangchon Ureongii (YU) rice makgeolli.

**Table 3:** Genotype-phenotype relationship of 4-VG production in the makgeolli isolates.

Strain	*FDC1* nucleotide	Fdc1 amino acid	4-VG production
S288C	160A, 232A (haploid)	Lys54, Lys78	+
CEN.PK2-1C	160A, 232A, Δ793-885 (haploid)	Lys54, Lys78, Δ265-295	−
CS-CAU12	160A/160A, 232A/232A (homozygous diploid)	Lys54/Lys54, Lys78/Lys78	+
CS-CAU26	160A/160A, 232A/232A (homozygous diploid)	Lys54/Lys54, Lys78/Lys78	+
HC-CAU28	160T/160T, 232A/232A (homozygous diploid)	Stop54/Stop54, Lys78/Lys78 (Premature termination at 54)	−
KY-CAU1	160A/160T, 232A/232A(heterozygous diploid)	Stop54/Lys54, Lys78/Lys78	−
KY-CAU18	160T/160T, 232A/232A (homozygous diploid)	Stop54/Stop54, Lys78/Lys78	−
YU-CAU13	160A/160A, 232T/232T (homozygous diploid)	Lys54/Lys54, Stop78/Stop78 (Premature termination at 78)	−

Position 160 or 232 in *FDC1* is changed to thymine (T) in the strains HC-CAU28, KY-CAU1, KY-CAU18, and YU-CAU13.
