# Supplementary material for: Exploration of Genomic Features and Volatile Flavor Production of Yeasts Isolated from Commercial Korean Rice Wine Makgeolli
Source: J Microbiol Biotechnol. 2026 Aug 3;36:e2605009. doi: 10.4014/jmb.2605.05009 (PMC13454834; doi:10.4014/jmb.2605.05009)
Supplement: Supplementary file 1 [file jmb-36-e2605009-supple.pdf]

J. Microbiol. Biotechnol. 2026. 36: e2605009

https://doi.org/10.4014/jmb.2605.05009

Supplementary Tables and Figures

Exploration of genomic features and volatile flavor production of yeasts isolated from commercial Korean rice wine makgeolli

Su Jin Yoo, Jongbeom Kim, Yeon Ju Park, Haeyoung Park, Hye Yun Moon, Sang Wan Han, Seon Woo Song, Seong-Il Eyun, Che Ok Jeon, and Hyun Ah Kang

Department of Life Science, Chung-Ang University, Seoul 06974, Republic of Korea

Contents:

Tables S1. Plasmids and primers used in this study

Tables S2. ITS sequence identity of yeast isolates obtained from commercial makgeolli products using BLAST ITS database

Figure S1. The internal transcribed spacer (ITS) sequence alignment.

Figure S2. Multiple alignment of amino acid sequences of the Pad1 and Fdc1 proteins.

Figure S3. Multiple alignment of nucleotide sequences of the *FDC1* gene of the makgeolli isolates.

Figure S4. Comparative volatile flavor profiling and qRT-PCR analysis of the genes involved in volatile ester and 4-VG biosynthesis.

Figure S5. Transcriptional regulation of the *FDC1* gene in the makgeolli isolates in response to ferulic acid.

Table S1. Plasmids and primers used in this study

Plasmid	Description	Reference
YCpUT-ScFDC1-HA	*S. cerevisiae* CEN vector with Amp^R, *URA3,* and the wild-type *FDC1* under the *TEF1* promoter and *GAL7* terminator	[16]
YCpNT-ScFDC1-HA	Derivatized from YCpUT-ScFDC1-HA by exchanging *URA3* with *NAT* as a selection marker	[16]
YCpNAT-C9U3g	*S. cerevisiae* CEN vector containing Amp^R, *NAT,* Cas9 under the *TEF1* promoter and *CYC1* terminator and *URA3* targeting gRNA	This study

Primer	Sequence (5'→3')	Description
ScAct1 Fw	gaaatgcaaaccgctgctca	qRT-PCR
ScAct1 Rv	cggcagattccaaacccaaa	
ScATF1_qRT-PCR_1F	gtgcgatcgtgccatcg	
ScATF1_qRT-PCR_2B	ccaaggaaaatgcttggtgcc	
ScATF2_qRT-PCR_1F	cgttcggcctaaacgtttgc	
ScATF2_qRT-PCR_2B	gcaaattcgccgatggtttgg	
ScIAH1_qRT-PCR_1F	ctgcacttttccggaaaaggg	
ScIAH1_qRT-PCR_2B	gatccatcatctagcacatctctcc	
ScEAT1_qRT-PCR_1F	caaaaaggcacagccagtcg	
ScEAT1_qRT-PCR_2B	ctcggacatcagcacaggg	
ScEEB1_qRT-PCR_1F	gcaacggatgatccagttacagg	
ScEEB1_qRT-PCR_2B	caagaactcggcagcttgc	
ScEHT1_qRT-PCR_1F	gggatgatcctgttgtcggc	
ScEHT1_qRT-PCR_2B	ccttggtagcccacgagttg	
ScILV2_qRT-PCR_1F	gcccagttttgcttgaagtgg	
ScILV2_qRT-PCR_2B	ccgcctgtacgcttatgacg	
ScBDH1_qRT-PCR_1F	gccaccaacattgcagtttgg	
ScBDH1_qRT-PCR_2B	ccgttgtggatggcacgaac	
ScPDC1_qRT-PCR_1F	gtcgctaccaccggtgaatg	
ScPDC1_qRT-PCR_2B	ggtagcagcagtcaacttagcttg	
ScPDC5_qRT-PCR_1F	gattcacggtcctcatgccg	
ScPDC5_qRT-PCR_2B	cccattcaccagtggtagcaac	
ScARO10_qRT-PCR_1F	gcattcggagacttcgacgg	
ScARO10_qRT-PCR_2B	cggggaaatccaattcgcc	
ScSCS7_qRT-PCR_1F	cgaatgtcatttcttcttgcaccac	
ScSCS7_qRT-PCR_2B	cttcgtcccaaaaccaggatg	
ScADL2_qRT-PCR_1F	gcaggaactgtttggatcaactc	
ScADL2_qRT-PCR_2B	ggtatcaacaccactttgccc	
ScADL6_qRT-PCR_1F	cgaaacagaatctttgagcacagg	
ScADL6_qRT-PCR_2B	ccagattgcttaacaccaccg	
FDC Fw	gaaccccggtctttgtacgg	
FDC1 Rv	gagcctgagcctcataaggc	
PAD1 Fw	ctggttactcgggaaacccc	
PAD1 Rv	ggtcatgaaggctcttgggtc	

Table S2. ITS sequence identity of yeast isolates obtained from commercial makgeolli products using BLAST ITS database

No.	Isolates	Identified Species (Percent identity)
1	KY-CAU #1	*S. cerevisiae* (99.31%)/*S. cariocanus* (98.76%)/*S. paradoxus* (98.66%)
2	CS-CAU #2	*S. cerevisiae* (99.18%)/*S. cariocanus* (98.64%)/*S. paradoxus* (98.51%)
3	CS-CAU #3	*S. cerevisiae* (99.18%)/*S. cariocanus* (98.64%)/*S.paradoxus* (98.51%)
4	CS-CAU #4	N/D
5	CS-CAU #5	*S. cerevisiae* (99.18%)/*S. cariocanus* (98.64%)/*S.paradoxus* (98.51%)
6	CS-CAU #6	N/D
7	CS-CAU #7	N/D
8	CS-CAU #8	N/D
9	CS-CAU #9	N/D
10	CS-CAU #10	*S. cerevisiae* (99.18%)/*S. cariocanus* (98.64%)/*S. paradoxus* (98.51%)
11	CS-CAU #11	N/D
12	CS-CAU #12	*S. cerevisiae* (99.18%)/*S. cariocanus* (98.64%)/*S. paradoxus* (98.51%)
13	YU-CAU #13	*S. cerevisiae* (99.31%)/*S. cariocanus* (98.76%)/*S. paradoxus* (98.66%)
14	HC-CAU #14	*S. cerevisiae* (99.18%)/*S. cariocanus* (98.64%)/*S. paradoxus* (98.51%)
15	HC-CAU #15	*S. cerevisiae* (99.18%)/*S. cariocanus* (98.64%)/*S. paradoxus* (98.51%)
16	HC-CAU #16	*S. cerevisiae* (99.18%)/*S. cariocanus* (98.64%)/*S. paradoxus* (98.51%)
17	HC-CAU #17	*S. cerevisiae* (99.18%)/*S. paradoxus* (98.54%)/*S. mikatae* (97.89%)
18	KY-CAU #18	*S. cerevisiae* (99.34%)/*S. cariocanus* (98.81%)/*S. paradoxus* (98.57%)
19	CS-CAU #19	N/D
20	CS-CAU #20	*S. cerevisiae* (99.18%)/*S. paradoxus* (98.54%)/*S. mikatae* (97.88%)
21	CS-CAU #21	*S. cerevisiae* (99.18%)/*S. cariocanus* (98.64%)/*S. paradoxus* (98.51%)
22	CS-CAU #22	*S. cerevisiae* (99.18%)/*S. paradoxus* (98.54%)/*S. mikatae* (97.88%)
23	CS-CAU #23	*S. cerevisiae* (99.18%)/*S. cariocanus* (98.64%)/*S. paradoxus* (98.51%)
24	CS-CAU #24	*S. cerevisiae* (99.27%)/*S. paradoxus* (98.86%)/*S. mikatae* (98.16%)
25	CS-CAU #25	N/D
26	CS-CAU #26	*S. cerevisiae* (99.28%)/*S. paradoxus* (98.87%)/*S. mikatae* (98.17%)
27	CS-CAU #27	*S. cerevisiae* (99.18%)/*S. paradoxus* (98.54%)/*S. mikatae* (97.88%)
28	HC-CAU #28	*S. cerevisiae* (99.18%)/*S. paradoxus* (98.54%)/*S. mikatae* (97.88%)

* N/D: Not determined because the ITS region was not amplified, or the ITS sequence was not read. Cheongju Sinseonju makgeolli (CS), Haechang (HC) raw makgeolli, Kuksundang Yetal (KY) makgeolli, and Yangchon Ureongii (YU) rice makgeolli.

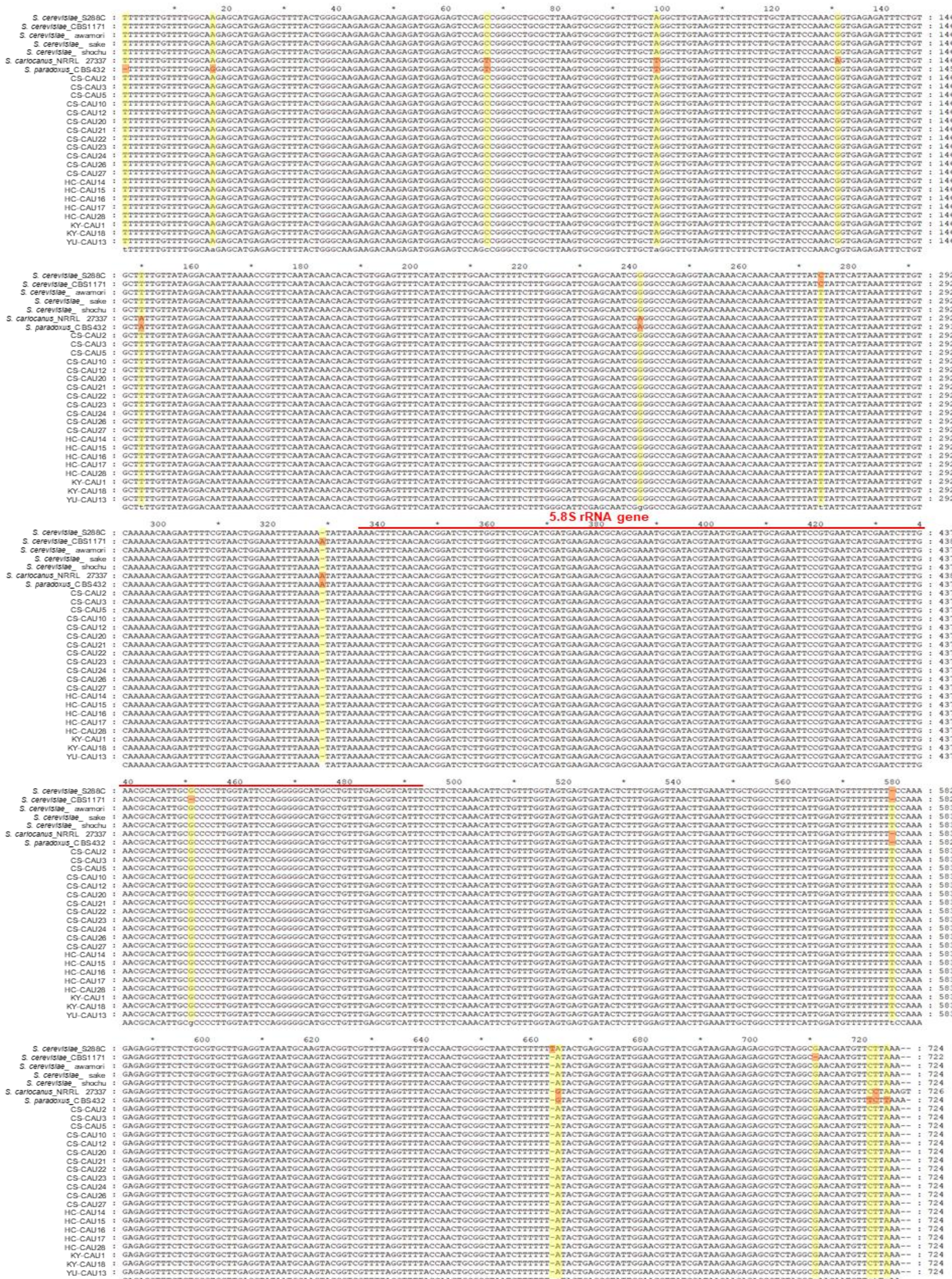

Fig. S1. The internal transcribed spacer (ITS) sequence alignment. The sequences of the ITS region of the makgeolli isolates, consisting of ITS1 and ITS2 separated by the 5.8S gene, were aligned with those of *S. cerevisiae* S288C reference strain (chromosome XII 455182-455932), CBS1171 type strain (NR_111007.1), awamori yeast S1 (LC576508.1), sake yeast Kyokai No.1

(LC576497.1), shochu yeast S18 (LC576525.1), *S. cariocanus* NRRL 27337 (NR_144772.1), *S. paradoxus* CBS 432 (NR_138272.1), and the makgeolli isolates, using the CLUSTALW method in MegAlign software and shaded using GeneDoc. The positions that exhibit different nucleotides are indicated as colored boxes. The chromosomal locus or GenBank accession number of the ITS sequences is indicated in parentheses.

A.

Pad1p

```
                  21                  41                  61                  81
S288C     MLLFPRRTNIAFFKTTGIFANFPLLGRTITTSPSFLTHKLSKEVTRASTSPPRPKRIVVAITGATGVALGIRLLQVLKELSVETHLVISK
CS-CAU12  MLLFPRRTNIAFFKTTGIFANFPLLGRTITTSPSFLTHKLSKEVTRVSTSPPRPKRIVVAITGATGVALGIRLLQVLKELSVETHLVISK
CS-CAU26  MLLFPRRTNIAFFKTTGIFANFPLLGRTITTSPSFLTHKLSKEVTRVSTSPPRPKRIVVAITGATGVALGIRLLQVLKELSVETHLVISK
HC-CAU28  MLLFPRRTNIAFFKTTGIFANFPLLGRTITTSPSFLTYKLSKEVTRVSTSPPRPKRIVVAITGATGVALGIRLLQVLKELSVETHLVISK
KY-CAU1   MLLFPRRTNIAFFKTTGIFANFPLLGRTITTSPSFLTYKLSKEVTRVSTSPPRPKRIVVAITGATGVALGIRLLQVLKELSVETHLVISK
KY-CAU18  MLLFPRRTNIAFFKTTGIFANFPLLGRTITTSPSFLTYKLSKEVTRVSTSPPRPKRIVVAITGATGVALGIRLLQVLKELSVETHLVISK
YU-CAU13  MLLFPRRTNIAFFKTTGIFANFPLLGRTITTSPSFLTYKLSKEVTRVSTSPPRPKRIVVAITGATGVALRIRLLQVLKELSVETHLVISK
          101                 121                 141                 161                 18
S288C     WGAATMKYETDWEPHDVAALATKTYSVRDVSACISSGSFQHDGMIVVPCSMKSLAAIRIGFTEDLITRAADVSIKENRKLLLVTRETPLS
CS-CAU12  WGAATMKYETDWEPHDVAALATKTYSVRDVSACISSGSFQHDGMIVVPCSMKSLAAIRIGFTEDLITRAADVSIKENRKLLLVTRETPLS
CS-CAU26  WGAATMKYETDWEPHDVAALATKTYSVRDVSACISSGSFQHDGMIVVPCSMKSLAAIRIGFTEDLITRAADVSIKENRKLLLVTRETPLS
HC-CAU28  WGAATMKYETDWEPHDVAALATKTYSVRDVSACISSGSFQHDGMIVVPCSMKSLAAIRIGFTEDLITRAADVSIKENRKLLLVTRETPLS
KY-CAU1   WGAATMKYETDWEPHDVAALATKTYSVRDVSACISSGSFQHDGMIVVPCSMKSLAAIRIGFTEDLITRAADVSIKENRKLLLVTRETPLS
KY-CAU18  WGAATMKYETDWEPHDVAALATKTYSVRDVSACISSGSFQHDGMIVVPCSMKSLAAIRIGFTEDLITRAADVSIKENRKLLLVTRETPLS
YU-CAU13  WGAATMKYETDWEPHDVAALATKTYSVRDVSACISSGSFQHDGMIVVPCSMKSLAAIRIGFTEDLITRAADVSIKENRKLLLVTRETPLS
          1                   201                 221                 241
S288C     SIHLENMLSLCRAGVIIFPPVPAFYTRPKSLHDLLEQSVGRILDCFGIHADTFPRWEGIKSK
CS-CAU12  SIHLENMLSLCRAGVIIFPPVPAFYTRPKSLHDLLEQSVGRILDCFGIHADTFPRWEGIKSK
CS-CAU26  SIHLENMLSLCRAGVIIFPPVPAFYTRPKSLHDLLEQSVGRILDCFGIHADTFPRWEGIKSK
HC-CAU28  SIHLENMLSLCRAGVIIFPPVPAFYTRPKSLHDLLEQSVGRILDCFGIHADTFPRWEGIKSK
KY-CAU1   SIHLENMLSLCRAGVIIFPPVPAFYTRPKSLHDLLEQSVGRILDCFGIHADTFPRWEGIKSK
KY-CAU18  SIHLENMLSLCRAGVIIFPPVPAFYTRPKSLHDLLEQSVGRILDCFGIHADTFPRWEGIKSK
YU-CAU13  SIHLENMLSLCRAGVIIFPPVPAFYTRPKSLHDLLEQSVGRILDCFGIHADTFPRWEGIKSK
```

B.

Fdc1p

```
                  21                  41                  61                  81
S288C     MRKLNPALEFRDFIQVLKDEDDLIEITEEIDPNLEVGAIMRRAYESHLPAPLFKNLKGASKDLFSILGCPAGLRSKEKGDHGRIAHHLGL
CS-CAU12  MRKLNPALEFRDFIQVLKDEDDLIEITEEIDPNLEVGAIMRRAYESHLPAPLFRNLKGASKDLFSILGCPAGLRSKEKGDHGRIAHHLGL
CS-CAU26  MRKLNPALEFRDFIQVLKDEDDLIEITEEIDPNLEVGAIMRRAYESHLPAPLFRNLKGASKDLFSILGCPAGLRSKEKGDHGRIAHHLGL
HC-CAU28  MRKLNPALEFRDFIQVLKDEDDLIEITEEIDPNLEVGAIMRRAYESHLPAPLF-NLKGASKDLFSILGCPAGLRSKEKGDHGRIAHHLGL
KY-CAU1   MRKLNPALEFRDFIQVLKDEDDLIEITEEIDPNLEVGAIMRRAYESHLPAPLF-NLKGASKDLFSILGCPAGLRSKEKGDHGRIAHHLGL
KY-CAU18  MRKLNPALEFRDFIQVLKDEDDLIEITEEIDPNLEVGAIMRRAYESHLPAPLF-NLKGASKDLFSILGCPAGLRSKEKGDHGRIAHHLGL
YU-CAU13  MRKLNPALEFRDFIQVLKDEDDLIEITEEIDPNLEVGAIMRRAYESHLPAPLFKNLKGASKDLFSILGCPAGLRSKK-GDHGRIAHHLGL
                                                   160th A→T (AAA→TAA)    232nd A→T (AAA→TAA)
          101                 121                 141                 161                 18
S288C     DPKTTIKEIIDYLLECKEKEPLPPITVPVSSAPCKTHILSEEKIHLQSLPTPYLHVSDGGKYLQTYGMWILQTPDKKWTNWSIARGMVVD
CS-CAU12  DPKTTIKEIIDYLLECKEKEPLPPITVPVSSAPCKTHILSEEKIHLQSLPTPYLHVSDGGKYLQTYGMWILQTPDKKWTNWSIARGMVVD
CS-CAU26  DPKTTIKEIIDYLLECKEKEPLPPITVPVSSAPCKTHILSEEKIHLQSLPTPYLHVSDGGKYLQTYGMWILQTPDKKWTNWSIARGMVVD
HC-CAU28  DPKTTIKEIIDYLLECKEKEPLPPITVPVSSAPCKTHILSEEKIHLQSLPTPYLHVSDGGKYLQTYGMWILQTPDKKWTNWSIARGMVVD
KY-CAU1   DPKTTIKEIIDYLLECKEKEPLPPITVPVSSAPCKTHILSEEKIHLQSLPTPYLHVSDGGKYLQTYGMWILQTPDKKWTNWSIARGMVVD
KY-CAU18  DPKTTIKEIIDYLLECKEKEPLPPITVPVSSAPCKTHILSEEKIHLQSLPTPYLHVSDGGKYLQTYGMWILQTPDKKWTNWSIARGMVVD
YU-CAU13  DPKTTIKEIIDYLLECKEKEPLPPITVPVSSAPCKTHILSEEKIHLQSLPTPYLHVSDGGKYLQTYGMWILQTPDKKWTNWSIARGMVVD
          1                   201                 221                 241                 261
S288C     DKHITGLVIKPQHIRQIADSWAAIGKANEIPFALCFGVPPAAILVSSMPIPEGVSESDYVGAILGESVPVVKCETNDIMVPATSEMVFEG
CS-CAU12  DKHITGLVIKPQHIRQIADSWAAIGKANEIPFALCFGVPPAAILVSSMPIPEGVSESDYVGAILGESVPVVKCETNDIMVPATSEMVFEG
CS-CAU26  DKHITGLVIKPQHIRQIADSWAAIGKANEIPFALCFGVPPAAILVSSMPIPEGVSESDYVGAILGESVPVVKCETNDIMVPATSEMVFEG
HC-CAU28  DKHITGLVIKPQHIRQIADSWAAIGKANEIPFALCFGVPPAAILVSSMPIPEGVSESDYVGAILGESVPVVKCETNDIMVPATSEMVFEG
KY-CAU1   DKHITGLVIKPQHIRQIADSWAAIGKANEIPFALCFGVPPAAILVSSMPIPEGVSESDYVGAILGESVPVVKCETNDIMVPATSEMVFEG
KY-CAU18  DKHITGLVIKPQHIRQIADSWAAIGKANEIPFALCFGVPPAAILVSSMPIPEGVSESDYVGAILGESVPVVKCETNDIMVPATSEMVFEG
YU-CAU13  DKHITGLVIKPQHIRQIADSWAAIGKANEIPFALCFGVPPAAILVSSMPIPEGVSESDYVGAILGESVPVVKCETNDIMVPATSEMVFEG
          281                 301                 321                 341                 36
S288C     TLSLTDTHLEGPFGEMHGYVFKSQGHPCPLYTVKAMSYRDNAILPVSNPGLCTDETHTLIGSLVATEAKELAIESGLPILDAFMPYEAQA
CS-CAU12  TLSLTDTHLEGPFGEMHGYVFKSQGHPCPLYTVKAMSYRDNAILPVSNPGLCTDETHTLIGSLVATEAKELAIESGLPILDAFMPYEAQA
CS-CAU26  TLSLTDTHLEGPFGEMHGYVFKSQGHPCPLYTVKAMSYRDNAILPVSNPGLCTDETHTLIGSLVATEAKELAIESGLPILDAFMPYEAQA
HC-CAU28  TLSLTDTHLEGPFGEMHGYVFKSQGHPCPLYTVKAMSYRDNAILPVSNPGLCTDETHTLIGSLVATEAKELAIESGLPILDAFMPYEAQA
KY-CAU1   TLSLTDTHLEGPFGEMHGYVFKSQGHPCPLYTVKAMSYRDNAILPVSNPGLCTDETHTLIGSLVATEAKELAIESGLPILDAFMPYEAQA
KY-CAU18  TLSLTDTHLEGPFGEMHGYVFKSQGHPCPLYTVKAMSYRDNAILPVSNPGLCTDETHTLIGSLVATEAKELAIESGLPILDAFMPYEAQA
YU-CAU13  TLSLTDTHLEGPFGEMHGYVFKSQGHPCPLYTVKAMSYRDNAILPVSNPGLCTDETHTLIGSLVATEAKELAIESGLPILDAFMPYEAQA
          1                   381                 401                 421                 441
S288C     LWLILKVDLKGLQALKTTPEEFCKKVGDIYFRTKVGFIVHEIILVADDIDIFNFKEVIWAYVTRHTPVADQMAFDDVTSFPLAPFVSQSS
CS-CAU12  LWLILKVDLKGLQALKTTPEEFCKKVGDIYFRTKVGFIVHEIILVADDIDIFNFKEVIWAYVTRHTPVADQMAFDDVTSFPLAPFVSQSS
CS-CAU26  LWLILKVDLKGLQALKTTPEEFCKKVGDIYFRTKVGFIVHEIILVADDIDIFNFKEVIWAYVTRHTPVADQMAFDDVTSFPLAPFVSQSS
HC-CAU28  LWLILKVDLKGLQALKTTPEEFCKKVGDIYFRTKV-FIVHEIILVADDIDIFNFKEVIWAYVTRHTPVADQMAFDDVTSFPLAPFVSQSS
KY-CAU1   LWLILKVDLKGLQALKTTPEEFCKKVGDIYFRTKV-FIVHEIILVADDIDIFNFKEVIWAYVTRHTPVADQMAFDDVTSFPLAPFVSQSS
KY-CAU18  LWLILKVDLKGLQALKTTPEEFCKKVGDIYFRTKV-FIVHEIILVADDIDIFNFKEVIWAYVTRHTPVADQMAFDDVTSFPLAPFVSQSS
YU-CAU13  LWLILKVDLKGLQALKTTPEEFCKKVGDIYFRTKVGFIVHEIILVADDIDIFNFKEVIWAYVTRHTPVADQMAFDDVTSFPLAPFVSQSS
                                       1,188th T deletion
                    461                 481                 501
S288C     RSKTMKGGKCVTNCIFRQQYERSFDYITCNFEKGYPKGLVDKVNENWKRYGYK
CS-CAU12  RSKTMKGGKCVTNCIFRQQYERSFDYITCNFEKGYPKGLVDKVNENWKRYGYK
CS-CAU26  RSKTMKGGKCVTNCIFRQQYERSFDYITCNFEKGYPKGLVDKVNENWKRYGYK
HC-CAU28  RSKTMKGGKCVTNCIFRQQYERSFDYITCNFEKGYPKGLVDKVNDNWKRYGYK
KY-CAU1   RSKTMKGGKCVTNCIFRQQYERSFDYITCNFEKGYPKGLVDKVNDNWKRYGYK
KY-CAU18  RSKTMKGGKCVTNCIFRQQYERSFDYITCNFEKGYPKGLVDKVNDNWKRYGYK
YU-CAU13  RSKTMKGGKCVTNCIFRQQYERSFDYKTCNFEKGYPKGLVDKVNENWKRYGYK
```

Fig. S2. Multiple alignment of amino acid sequences of the Pad1 and Fdc1 proteins. The multiple amino acid sequence alignment of the Pad1 **(A)** and Fdc1 **(B)** proteins from *S. cerevisiae* S288C and the makgeolli isolates (CS-CAU12, CS-CAU26, HC-CAU28, KY-CAU1, KY-CAU18, and YU-CAU13) based on their whole genome sequences. Residues that are not conserved across

all sequences are highlighted in grey and in black. Nonsense mutations and nucleotide deletions are indicated in red.

A

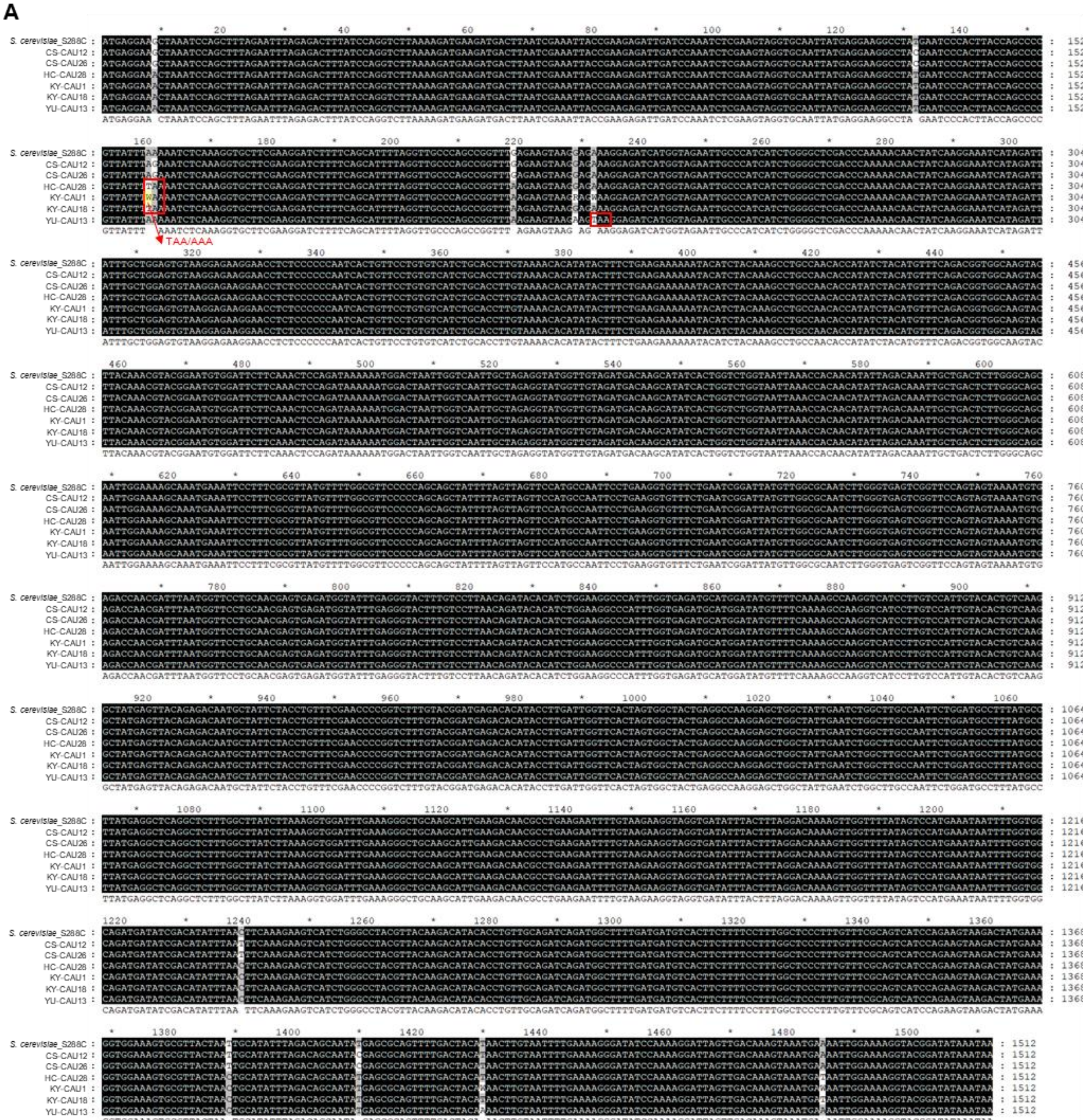

B

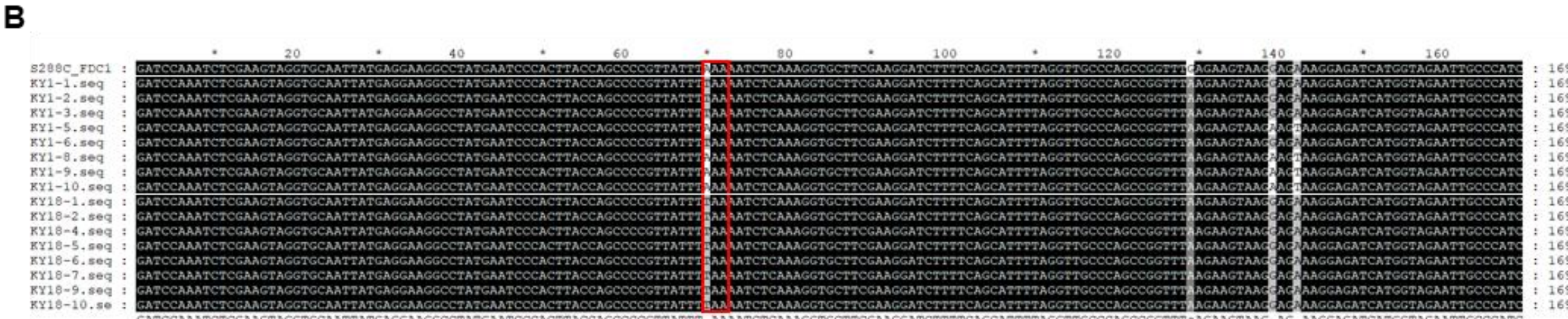

Fig. S3. Multiple alignment of nucleotide sequences of the *FDC1* gene of the makgeolli isolates. (A) The multiple nucleotide sequence alignment of the *FDC1* gene from *S. cerevisiae* S288C and the makgeolli isolates (CS-CAU12, CS-CAU26, HC-CAU28, KY-CAU1, KY-CAU18, and YU-CAU13) based on their whole genome sequences. **(B)** Sanger sequencing analysis for the determination of zygosity. Partial *FDC1* fragments encompassing the mutation site from KY-CAU1 (KY1) and KY-CAU18 (KY18) were amplified, cloned to T-vectors, and sequenced.

Residues that are not conserved across all sequences are highlighted in grey and in white. Nonsense mutation sites are indicated in red.

A.

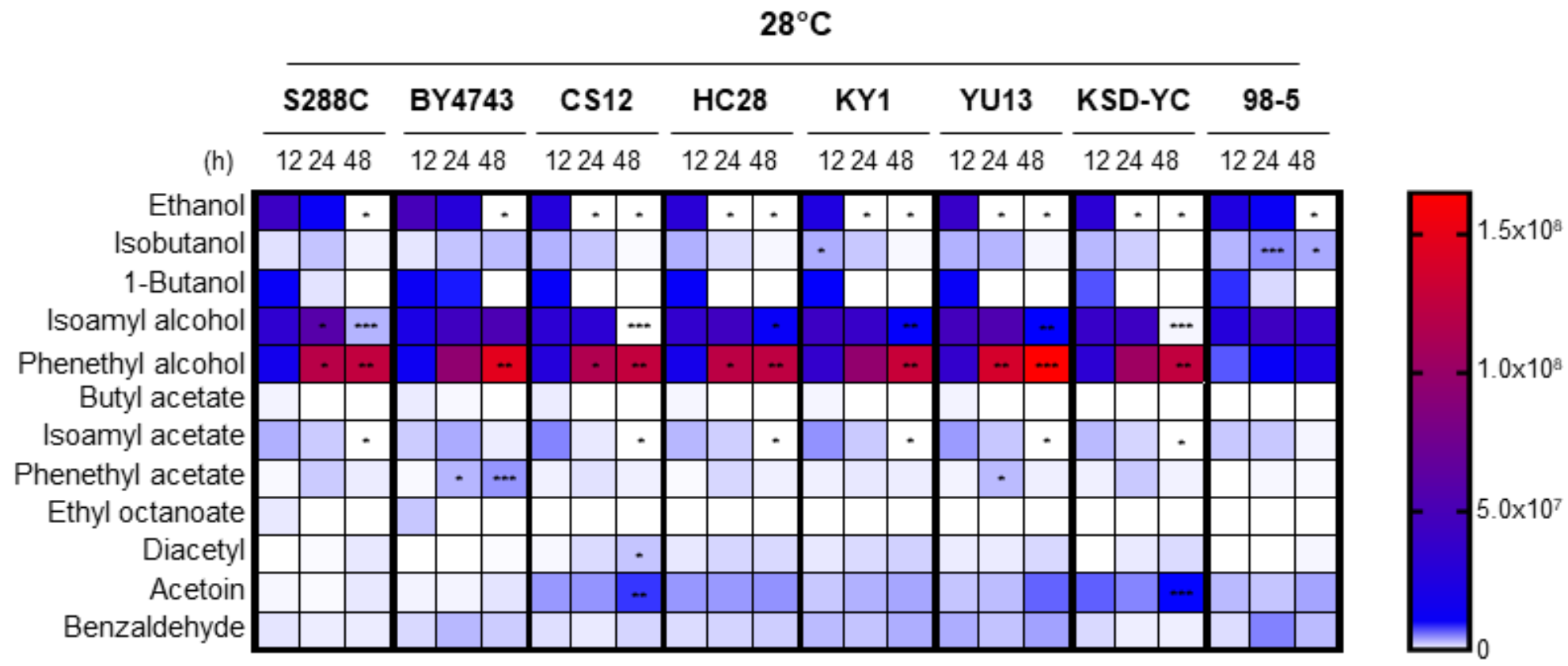

B.

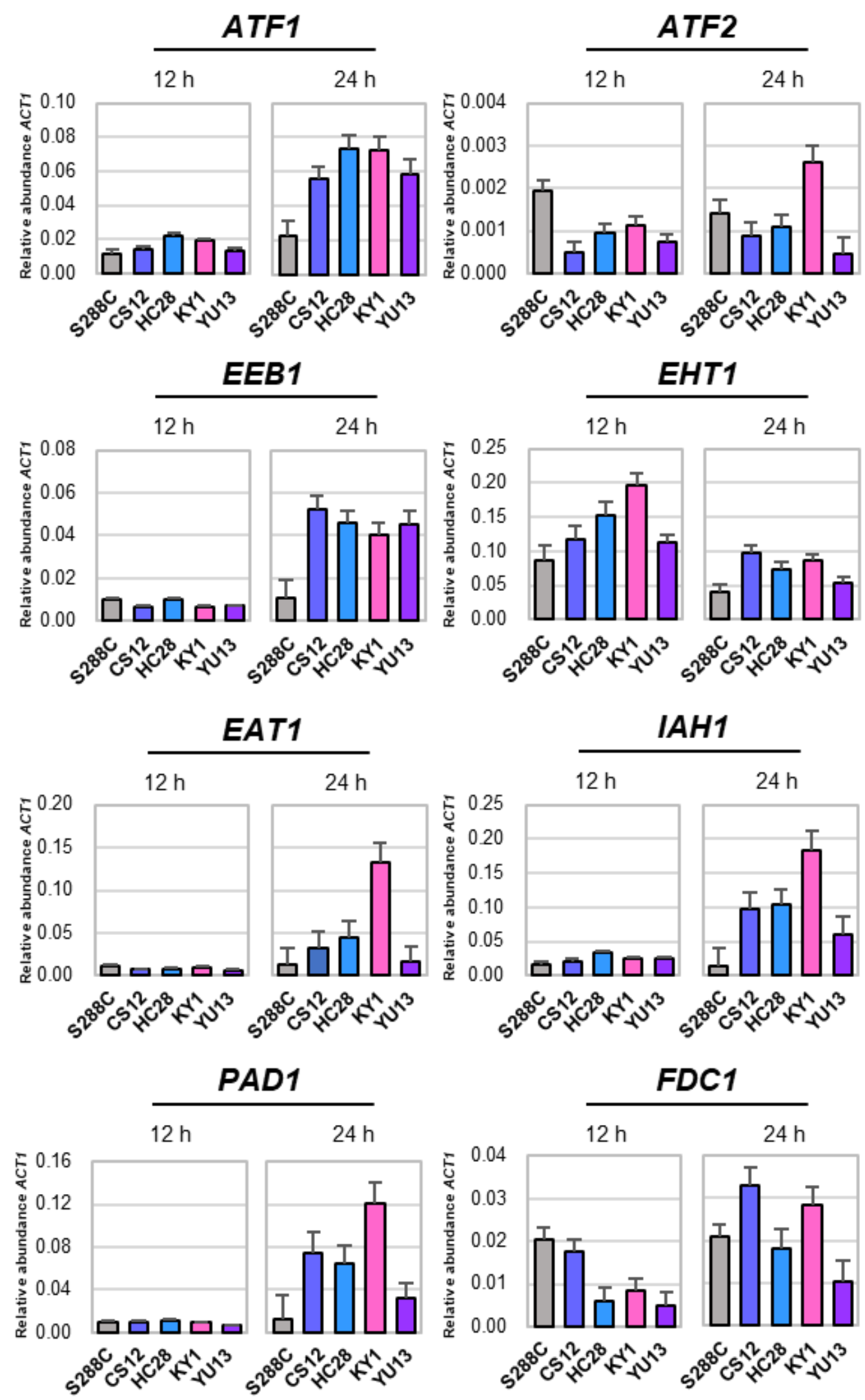

Fig. S4. **Comparative volatile flavor profiling and qRT-PCR analysis of the genes involved in volatile ester and 4-VG biosynthesis. (A)** Comparison of the volatile flavor profiles of the makgeolli isolates with those of *S. cerevisiae* S288C reference strain, BY4743 diploid lab strain, and the two diploid industrial strains, KSD-YC and 98-5, used for fermentation of commercial makgeolli, based on HS-SPME GC/MS. GraphPad Prism (ver. 8.4) was used to generate the plots.

*, $p < 0.05$; **, $p < 0.01$; ***, $p < 0.001$; and ****, $p < 0.0001$, between cell cultures and culture of S288C at 12 h (28°C) (one-way ANOVA). **(B)** The transcript levels of the *ATF1*, *ATF2*, *IAH1*, *EEB1*, *EHT1*, and *EAT1* genes involved in the formation of volatile esters and the *PAD1* and *FDC1* genes involved in 4-VG formation at 12 h and 24 h are shown as relative ratios to that of the *ACT1* gene. Error bars represent the standard error of the mean from biological duplicate measurements.

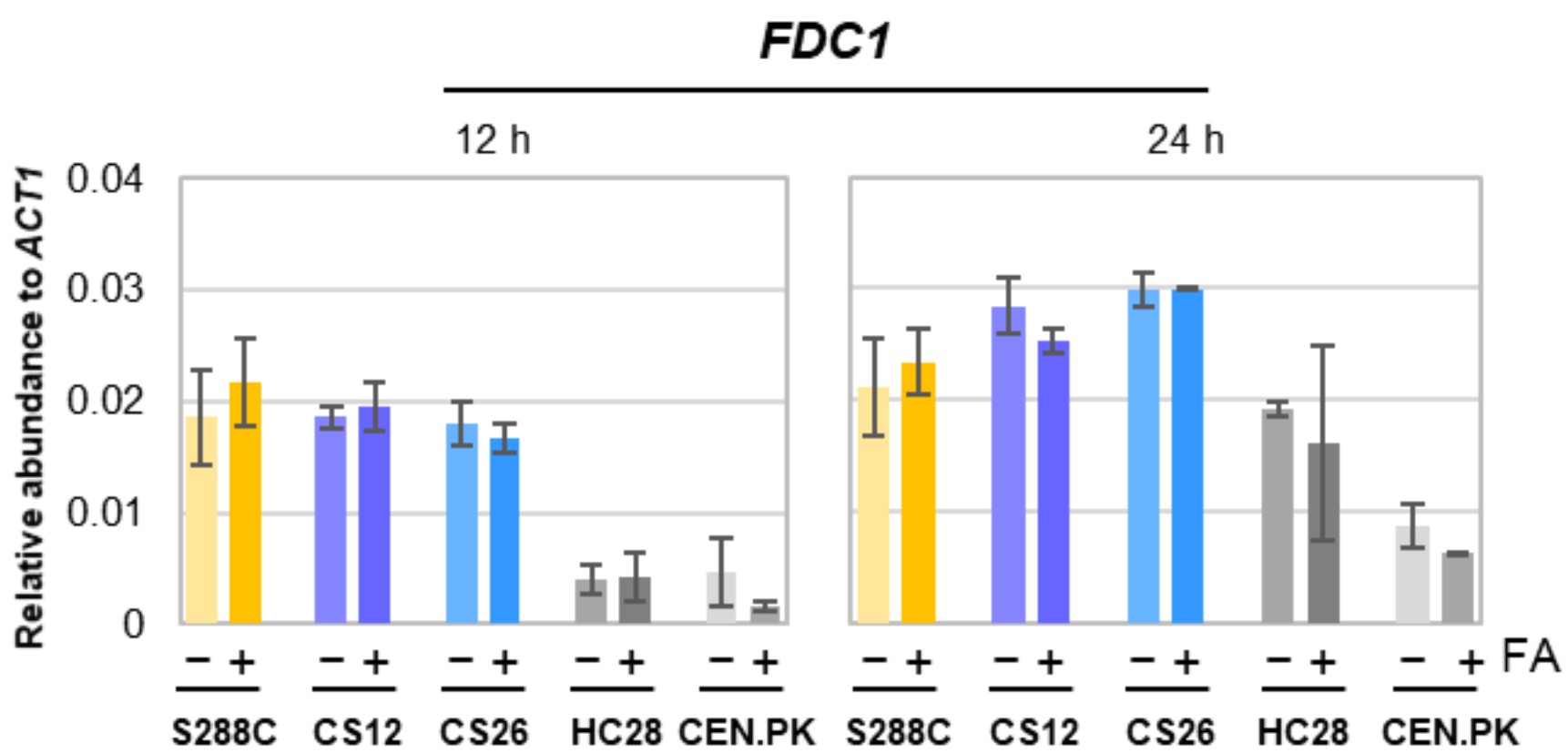

Fig. S5. **Transcriptional regulation of the *FDC1* gene in the makgeolli isolates in response to ferulic acid.** The relative transcript levels of the *FDC1* gene were analyzed by qRT-PCR in the 4-VG-producing makgeolli isolates (CS-CAU12 and CS-CAU26), along with the S288C reference strain as a positive control, and the strains HC-CAU28 and CEN.PK2-1C as negative controls. The yeast cells were cultivated in YPD medium with (+) or without (−) ferulic acid (FA) at 28°C and samples were collected at 12 h and 24 h. The expression levels of *FDC1* are shown as relative abundance to that of *ACT1*. Error bars represent the standard error or the mean from biological duplicate measurements.
